# Genomic Analysis Uncovers the Prognostic and Immunogenetic Feature of Pyroptosis in Gastric Carcinoma: Indication for Immunotherapy

**DOI:** 10.3389/fcell.2022.906759

**Published:** 2022-07-13

**Authors:** Jie Zhou, Run-cong Nie, Yi-xin Yin, Yun Wang, Shu-qiang Yuan, Zi-han Zhao, Xin-ke Zhang, Jin-ling Duan, Ying-bo Chen, Zhi-wei Zhou, Dan Xie, Yuan-fang Li, Mu-yan Cai

**Affiliations:** ^1^ State Key Laboratory of Oncology in South China, Collaborative Innovation Center for Cancer Medicine, Sun Yat-sen University Cancer Center, Guangzhou, China; ^2^ Department of Gastric Surgery, Sun Yat-sen University Cancer Center, State Key Laboratory of Oncology in South China, Collaborative Innovation Center for Cancer Medicine, Guangzhou, China; ^3^ Department of Hematologic Oncology, Sun Yat-sen University Cancer Center, State Key Laboratory of Oncology in South China, Collaborative Innovation Center for Cancer Medicine, Guangzhou, China; ^4^ Department of Pathology, Sun Yat-sen University Cancer Center, State Key Laboratory of Oncology in South China, Collaborative Innovation Center for Cancer Medicine, Guangzhou, China

**Keywords:** pyroptosis, immune, prognosis, immunotherapy, gastric cancer

## Abstract

Crosstalk between pyroptosis and tumor immune microenvironment (TIME) in cancer has yet to be elucidated. Herein, we aimed to explore the role of pyroptosis and its association with TIME in gastric cancer. Unsupervised clustering was performed to identify the pyroptosis-related clusters. Pyroptosis risk score was constructed using LASSO Cox regression. Clinicopathological and genetic data of pyroptosis clusters and pyroptosis risk scores were explored. Reproducibility of pyroptosis risk score in predicting response to immunotherapy and screening potential antitumor drugs was also investigated. Three pyroptosis clusters with distinct prognosis, immune cell fractions and signatures, were constructed. A low-pyroptosis risk score was characterized by increased activated T-cell subtype and M1 macrophage, decreased M2 macrophage, higher MSI status, and TMB. Meanwhile, low-score significantly correlated with PD-L1 expression, antigen presentation markers, and IFN-γ signature. The 5-year AUCs of PRS were 0.67, 0.62, 0.65, 0.67, and 0.67 in the TCGA, three external public and one real-world validation (SYSUCC) cohorts. Multivariable analyses further validated the prognostic performance of the pyroptosis risk scoring system, with HRs of 2.43, 1.83, 1.78, 2.35, and 2.67 (all *p* < 0.05) in the five cohorts. GSEA indicated significant enrichment of DNA damage repair pathways in the low-score group. Finally, the pyroptosis risk scoring system was demonstrated to be useful in predicting response to immunotherapy, and in screening potential antitumor drugs. Our study highlights the crucial role of interaction between pyroptosis and TIME in gastric cancer. The pyroptosis risk scoring system can be used independently to predict the survival of individuals and their response to immunotherapy.

## GRAPHICAL ABSTRACT

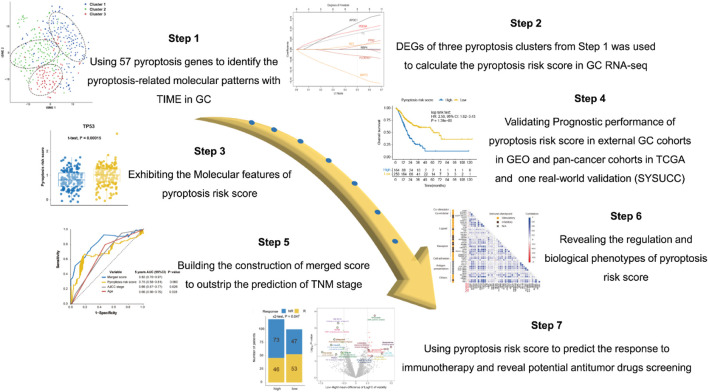

## Introduction

Gastric cancer (GC) is the second leading cause of cancer mortality worldwide, and it was responsible for about 782,685 deaths in 2018 (Bray et al., 2018). The majority of pathological types of GCs are adenocarcinomas, which can be further divided into intestinal and diffuse types according to the Lauren classification (Laurén, 1965), or various other subtypes (i.e., tubular, papillary, signet ring cell, and hepatoid type) according to the WHO classification (WHO, 2019). Hitherto, surgical or endoscopic resection is the mandatory treatment for early or localized GC. However, approximately 30–40% of GC patients experience relapse or metastasis after curative resection (Sasako et al., 2011; Noh et al., 2014). It is worth noting that the prognosis of patients with similar clinicopathological features (i.e., age, Lauren subtype, and TNM stage) and therapeutic strategies could vary significantly, indicating that substantial heterogeneity exists among GC and that the prognostic performance of the TNM staging system should be scrutinized. Furthermore, despite having been proven to improve the survival of patients with metastatic or inoperable GC (Koizumi et al., 2008; Yamada et al., 2015; Kang et al., 2020), the therapeutic effect of cytotoxic drugs is still far below the expectation of patients and clinicians. The latest evidence has demonstrated that blockade of programmed cell death protein 1 (PD-1) is non-inferior to chemotherapy (Shitara et al., 2020a), and the combination of PD-1 inhibitor with chemotherapy shows superior survival versus chemotherapy alone in patients with advanced GC (Janjigian et al., 2021). Nonetheless, only 15–60% of patients respond to anti-PD-1 immunotherapy. Therefore, the identification of novel markers with higher prognostic and predictive performance for GC is crucial in the clinical settings.

Pyroptosis, a form of gasdermin-mediated cell death, attracted researchers’ attention as it plays a role in innate immunity and diseases (Ruan et al., 2020; Yu et al., 2021). In response to infection or other immunological threats, the cells assemble pro-caspases and inflammasome sensors to form inflammasomes and activated caspases. Cleaved-caspase 1/4/5 subsequently cleaves pro-IL-1β/18 and gasdermins (GSDMs). Proteolytic cleavage of the GSDMs allows the N-terminal (NT) domain to oligomerize and form pores in the cell membranes. GSDMs pores can induce membrane to disrupt and trigger inflammatory cell death through the release of pro-inflammatory cytokines (i.e., IL-1β and IL-18) to the extracellular space (Zheng et al., 2021). At the time of this writing, Zhang et al. revealed that GSDME (also known as DFNA5) can inhibit tumor growth by enhancing phagocytosis of macrophages, as well as increasing the number and functions of natural-killer (NK) cells and cytotoxic T lymphocytes (Zhang et al., 2020). Moreover, Ye et al. reported a pyroptosis-related gene signature that reflects tumor immunity and predicts the prognosis of ovarian cancer (Ye et al., 2021). These findings suggest that pyroptosis may play an important role in the tumor immune microenvironment (TIME). In addition, the immune subsets within the TIME also affect the efficiency of the immune system, such as T cells (Ayers et al., 2017; Zhang et al., 2020), NK cells (Havel et al., 2019), and even eosinophils (Rossi et al., 2019). However, the effects and patterns of pyroptosis and its relationship with TIME in GC remain to be elucidated.

In this study, the pyroptosis patterns, as well as their association with the TIME features in GC, were investigated. Furthermore, we proposed a pyroptosis-based risk score to quantify the risk degree of pyroptosis. The pyroptosis risk score demonstrates robust prognostic performance for predicting survival and response to immunotherapy, suggesting that it may be used to assist clinicians in providing more efficient and personalized treatment for GC patients.

## Materials and Methods

### Gastric Cancer Cohorts and Data Processing

RNA-sequencing (RNA-seq) and clinicopathological data of GC and pan-cancer patients of The Cancer Genome Atlas (TCGA) were downloaded from the UCSC Xena database (https://xenabrowser.net/datapages/). We retrieved the datasets of GC from Gene Expression Omnibus (GEO) that meet the following criteria: 1) > 100 subjects; 2) overall survival data available; 3) the mRNA expression of targeted genes available. The raw data of GEO cohorts were downloaded and normalized using the multiarray quantile method (limma package). The expression matrix of TCGA and GEO data was normalized to transcripts per kilobase million (TPM) format and to use the sva package to adjust the potential batch effect. Genomic data were downloaded from the cBioPortal database (https://www.cbioportal.org/), and analyzed using the maftools package (Mayakonda et al., 2018). Tumor mutational burden (TMB) is calculated as the total number of somatic mutations divided by the full length of exons. The utilization of TCGA and GEO complied with the Declaration of Helsinki.

### Pyroptosis-Based Consensus Clustering Analysis

Searching with the keyword “pyroptosis” in Gene Set Enrichment Analysis (GSEA) (http://www.gsea-msigdb.org/gsea/index.jsp), 27 pyroptosis-related genes (PRGs) were identified from the gene set “REACTOME_PYROPTOSIS”. In addition, other PRGs were extracted from prior reviews (Green, 2019; Ruan et al., 2020; Bertheloot et al., 2021; Yu et al., 2021). In summary, a total of 57 PRGs were identified (Supplementary Table S1). These PRGs include 8 CHMP families, 7 caspases, 7 cytokines, 7 TLR families, 6 gasdermin superfamilies, 3 inflammasomes-mediated genes, 2 granzyme-mediated genes, and 17 un-assorted genes.

Unsupervised clustering based on mRNA expression of the 57 PRGs was used to identify different pyroptosis-related clusters using the ConsensusClusterPlus package (Wilkerson and Hayes, 2010). T-distributed stochastic neighbor embedding (t-SNE), a non-linear technique for dimensionality reduction, was applied to divide patients into distinct pyroptosis clusters based on the expression profiles of the PRGs (Cieslak et al., 2020). The levels of pyroptosis core genes (*CASP1*/*CASP4*/*CASP5*/*GSDMD*/*IL1B*/*IL18*) in the pyroptosis-related clusters were compared using Kruskal–Wallis test.

### Tumor Immune Microenvironment

The tumor immune contents for each patient in the TCGA cohort were estimated using the CIBERSORTx algorithm, which can infer a total of 22 immune cell fractions through cell-type-specific gene expression of bulk tissue RNA profiles (Newman et al., 2019). Only subjects with an empirical *p* value of less than 0.05 were eligible for further analysis. In addition, gene sets for nine immune signatures and immune checkpoints were obtained from previously described methods (Thorsson et al., 2018), and the single sample gene set enrichment analysis (ssGSEA) was applied to quantify the immune signatures using the GSVA package (Hänzelmann et al., 2013).

### Dimension and Pyroptosis Risk Score

The pyroptosis risk score is generated using the following four steps: 1) Screening for pyroptosis related differentially expressed genes (DEGs): GC patients from TCGA were divided into pyroptosis clusters and DEGs among the clusters were calculated with the limma package (Ritchie et al., 2015). Genes with adjusted *p* < 0.05 and absolute values of fold change >2 were considered pyroptosis-related DEGs. 2) Selecting prognostic pyroptosis-related DEGs: pyroptosis-related DEGs with *p* < 0.05 in the univariate Cox regression analysis were chosen. 3) Constructing pyroptosis risk score: the least absolute shrinkage and selection operator (LASSO) Cox regression was applied to determine the optimal weighting coefficient and to construct a pyroptosis risk score based on the penalized maximum likelihood estimator using the glmnet package. The pyroptosis risk score for each patient of the TCGA and GEO cohorts was calculated with the following formula: pyroptosis risk score = ∑exp(i) × coef(i), where exp(i) represents the expression of an eligible gene and coef(i) represents the corresponding coefficient in the LASSO model. 4) Verifying robustness of the pyroptosis risk score: the correlation between pyroptosis risk score and pyroptosis core genes was calculated.

### Gene set Enrichment Analysis

Based on the KEGG gene sets (c2.cp.kegg.v7.4.symbols.gmt), GSEA was applied to identify the enrichment pathways between the high- and low-pyroptosis risk score groups using the clusterProfiler package. Pathways with a false discovery rate (FDR) adjusted *p* < 0.05 were considered significantly enriched.

### Immune Checkpoint Blockade Cohorts

Kim et al. reported on a PD1 inhibition cohort of metastatic GC (Kim et al., 2018), the RNA-seq and clinical data of which were obtained from the TIDE website (http://tide.dfci.harvard.edu/); however, the survival data were unavailable. Three anti-PD1 therapy cohorts of melanoma patients were included to validate the prognostic value of pyroptosis risk score: RNA-seq and clinical data of PRJEB23709 (Gide et al., 2019) and GSE100797 (Lauss et al., 2017) cohorts were acquired from the TIDE website, while data of the Liu cohort (Liu et al., 2019) were extracted from the supplementary data of the published article. The definition of overall survival (OS), recurrence-free survival (RFS), and progression-free survival (PFS) was described in the corresponding cohorts. Patients with complete or partial responses were considered responders. The pyroptosis risk score for patients in the ICB cohorts is calculated with the aforementioned formula of LASSO regression.

### Drug Sensitivity Prediction

The profiling relative inhibition simultaneously in the matrix (PRISM) drug repurposing resource (https://depmap.org/repurposing) contains a total of 4518 antitumor drugs across 578 human cancer cell lines (Corsello et al., 2020). The RNAs-seq data of 19 GC cell lines were acquired from the DepMap Portal (https://depmap.org/portal/). The pyroptosis risk score of the GC cell lines was calculated using the formula of LASSO regression, and then divided into the high- and low-score groups according to their median values. The drug sensitivity of an antitumor agent was quantified as log2 (fold change) of viability values relative to DMSO. Furthermore, the differences in drug sensitivity between cell lines with high- and low-pyroptosis risk scores were evaluated.

### Quantitative Real-Time RT-PCR Analysis and Survival Analysis for Eight Differential Expression Genes in Sun Yat-Sen University Cohort

According to LASSO regression, eight DEGs were identified. The prognostic values of these eight genes were verified through qRT-PCR. qRT-PCR analyses of eight DEGs were carried out on the tumor and the corresponding mucosal tissues. All these tissues were fresh and stored at −80°C in the Biological Specimen Bank of our institute before use. For tumor sample analysis, we received informed consent from each patient and approval from the Institutional Review Board of Sun Yat-sen University Cancer Center. The inclusion criteria were as follows: 1) having no adjuvant treatment before operation; 2) complete resection of the tumor; 3) incised margin was negative; 4) without distant metastasis; 5) follow-up data was detailed and complete.

The total RNA of the tissue was extracted using TRIzol reagent (Thermo Fisher Scientific, Waltham, MA, United States), and the concentration was detected using NanoDrop2000 Spectrophotometer (Thermo Scientific, Wilmington, DE, United States). Total RNA (1 μg) was reverse-transcribed and qPCR was performed using an SYBR Green reaction mix (Takara Biotechnology, Shiga, Japan) with an ABI Quant Studio5 Real-Time PCR System (Applied Biosystems, Carlsbad, CA, United States). The primer sequences were shown in Supplementary Table S3. Glyceraldehyde 3-phosphate dehydrogenase (GAPDH) was used for normalization. Only the high-quality tissues (CT_GAPDH_RNA_: 15–21 and CT_targeted_RNA_ < 40) were included. The expression of individual RNA molecules was determined by the -△CT approach (△CT = CT_targeted_RNA_—CT_GAPDH_RNA_).

### Statistical Analysis

T-test, ANOVA, and Kruskal–Wallis test were employed to quantify the difference of continuous variables between two groups, multi-groups, and multiple comparisons, respectively. The Pearson correlation test was applied to quantify the association between two continuous variables. Comparisons of categorical factors were assessed by the chi-squared test. The cut-off values of categories in survival analysis were determined by the survminer package with a minimal of 15% of each subgroup. Survival was estimated using the Kaplan-Meier method and compared by log-rank test. The prognostic efficacy of pyroptosis risk score was assessed by time-dependent receiver operating characteristics curves (ROC) with the area under curve (AUC) using the timeROC package. The differences in AUC among variables were calculated using the bootstrap method. Uni- and multivariate cox regression analyses were employed to evaluate prognostic performance. A nomogram was constructed to visualize the prognostic values of entered factors in multivariate cox regression, and was used to establish the merged score. All the statistical analyses were performed by R software (version 4.1.0). Two-sided *p-*value less than 0.05 was considered statistically significant.

## Results

### Pyroptosis-Related Molecular Patterns With Tumor Immune Microenvironment in Gastric Cancer

**FIGURE 1:**
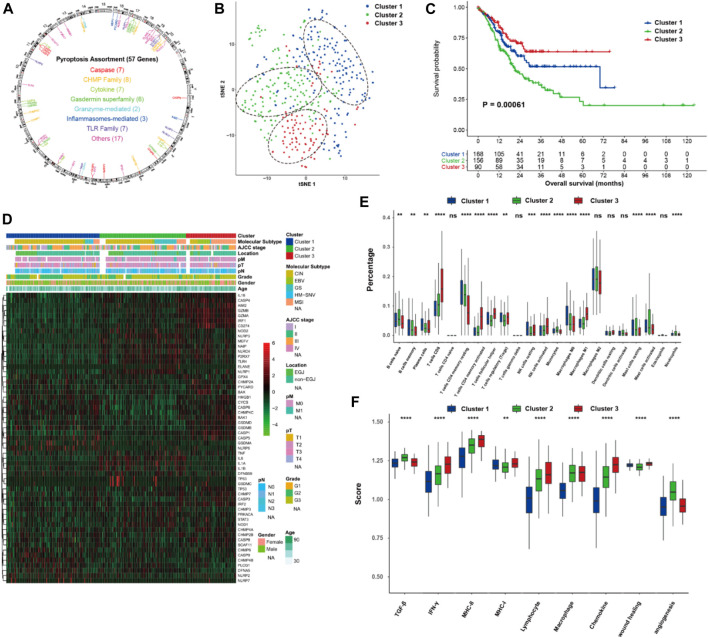
Pyroptosis-related molecular patterns with tumor immune microenvironment in gastric cancer. **(A)** The location of CNV of 57 PRGs on chromosomes. **(B)** t-SNE of gastric cancer based on the mRNA expression of 57 PRGs. **(C)** Kaplan-Meier curves of overall survival of the three pyroptosis clusters of gastric cancer. **(D)** Heatmap showing the correlations between pyroptosis clusters and clinicopathological characteristics, and the mRNA expression of 57 PRGs. **(E,F)** proportion of the 22 immune cell fractions and immune signatures among the pyroptosis clusters. PRGs, pyroptosis-related genes; ns: not significant; *p < 0.05; **p < 0.01; ***p < 0.001; ****p < 0.0001.

Overall, 57 PRGs were identified in this study. The location of CNV of these PRGs on chromosomes is depicted in Figure 1A. Among these PRGs, *TP53* exhibits the highest mutated frequency, followed by TLR4, CASP8, and NLRP3 (Supplementary Figure S1). To investigate the pyroptosis-related molecular patterns, we performed an unsupervised clustering analysis based on the mRNA expression of 57 PRGs and identified three distinct clusters in the TCGA data (i.e., Cluster 1, 2, and 3) (Figure 1B). Correlations between clusters and the expression of pyroptosis core genes indicated that Cluster 3 could be associated with the highest pyroptosis level (Supplementary Figure S2). Of the 3 pyroptosis clusters, Cluster 3 exhibited the most prolonged OS (*p* < 0.001; Figure 1C). Of note, Cluster 3 also showed higher percentage of microsatellite instability (MSI) subtype (7.2 vs. 11.7% vs. 54.2% for Cluster 1, 2, and 3, *p* < 0.001; Figure 1D), while the distributions of pathological T stage (*p* = 0.385), N stage (*p* = 0.360), and M stage (*p* = 0.194) were not different among the clusters. After dividing Cluster 3 into MSI-H and MSI-L/MSS subgroups, the MSI-H group exhibited a tendency of having more prolonged OS, but it did not reach a statistical difference (*p* = 0.22, Supplementary Figure S1C). Furthermore, the TIME features were explored using the CIBERSORTx and ssGSEA algorithms. Significantly higher proportions of CD8 T cells, activated CD4 memory T cells, activated NK cells, and M1 macrophages were observed in Cluster 3 (Figure 1E). The immune signature analysis also indicated that Cluster 3 corresponded with an inflamed cancer-immune phenotype (Figure 1F). In addition, we further analyzed the immune signatures in Cluster 3 with MSI-H or MSI-L/MSS. Notably, 5/22 immune cell fractions exhibited significant differences between two subgroups (Supplementary Figure S1D).

**FIGURE 2:**
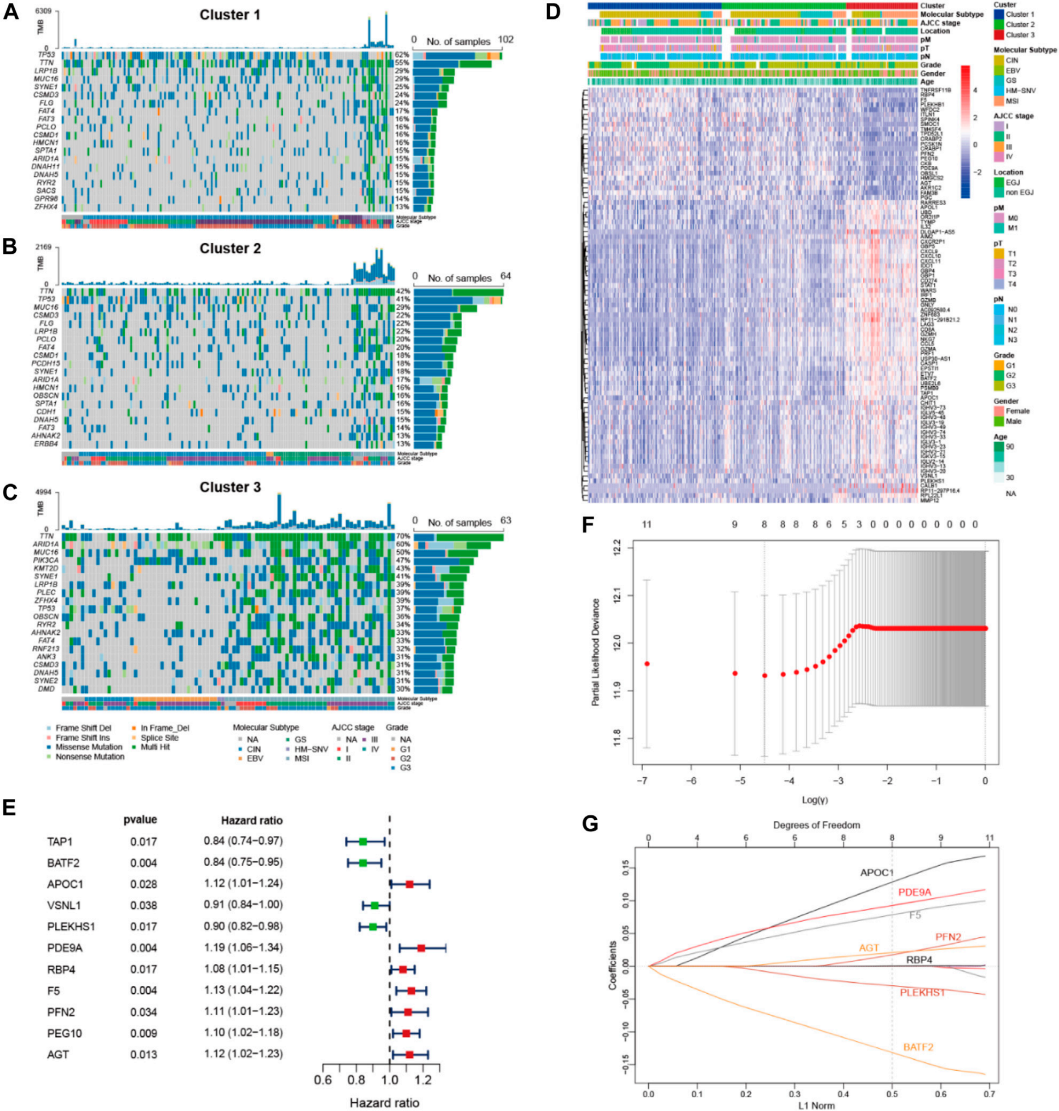
Genomic analysis of pyroptosis clusters and construction of pyroptosis risk scores. **(A,B,C)** Top 20 most frequently mutated genes of the 3 pyroptosis clusters. **(D)** Heatmap showing the correlations between pyroptosis clusters and DEGs, among which 62 genes and 23 genes were up- and down-regulated in pyroptosis cluster 3, respectively. **(E)** Forest plot of HRs for 11 DEGs. **(F)** Partial likelihood deviance of 11 DEGs identified by the LASSO regression model, in which 1000-fold cross-validation was used for variables selection. **(G)** LASSO coefficients of DEGs, each curve represents a gene. DEGs, differential expression genes; HRs, hazard ratios.

Figures 2A–C visualize the top 20 somatic mutations in these pyroptosis clusters. A higher frequency of somatic mutations with higher TMB (*p* < 0.001) was observed in Cluster 3. As compared with Cluster 1 and Cluster 2, Cluster 3 also showed higher mutation rates in *ARID1A*, *PIK3CA* and *KMT2D*, and a relatively lower mutation rate in *TP53*.

### Construction of Pyroptosis Risk Score

Transcriptomic expression profiles were compared among the three pyroptosis clusters of the TCGA data. A total of 85 DEGs were identified in Cluster 3, including 62 up-regulated and 23 down-regulated genes (Figure 2D). Among these DEGs, 11 exhibited associations with overall survival (Figure 2E). Next, these prognostic DEGs were analyzed using LASSO Cox regression to construct a pyroptosis risk score (Figures 2F,G). A total of 8 DEGs were identified to calculate the pyroptosis risk score, as follows:

Pyroptosis risk score = (0.145 × expression of *APOC1*) + (0.100 × expression of *PDE9A*) + (0.086 × expression of *F5*) + (0.025 × expression of *PFN2*) + (0.024 × expression of *AGT*) + (0.001 × expression of *RBP4*)—(0.033 × expression of *PLEKHS1*)—(0.146 × expression of *BATF2*).

We also identified the negative correlation between pyroptosis risk score and the expression of pyroptosis core genes (Supplementary Figure S3), indicating that the pyroptosis risk score could, in fact, represent the risk for pyroptosis. The pyroptosis risk scores for all the eligible cohorts in this study were calculated using the above formula.

### Molecular Features of Pyroptosis Risk Score

**FIGURE 3:**
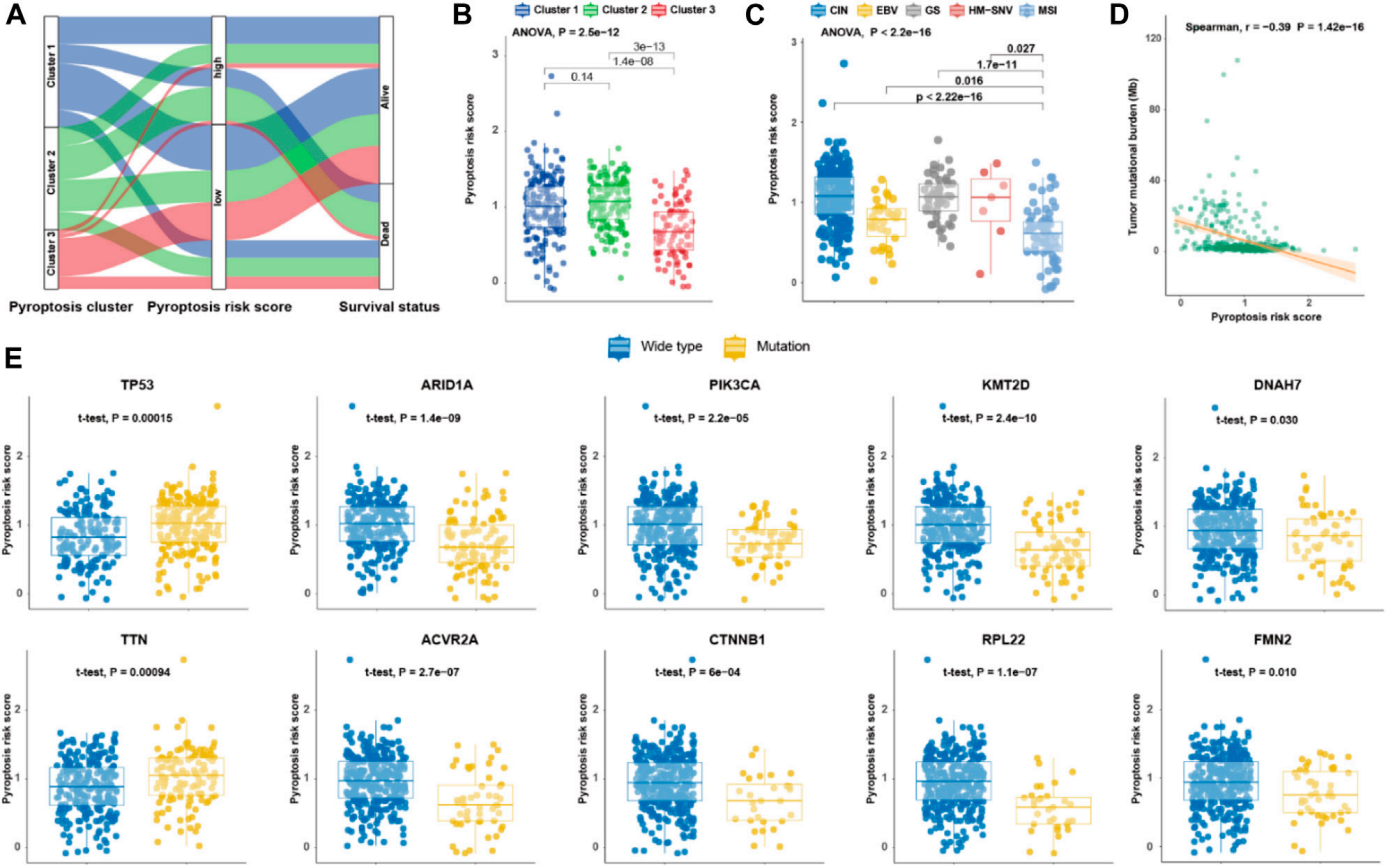
Molecular features of pyroptosis risk score for gastric cancer. **(A)** Alluvial diagram of pyroptosis clusters, pyroptosis risk score, and survival status. **(B,C)** Dot plot depicting the pyroptosis risk score in the three pyroptosis clusters, and TCGA molecular subtypes, respectively. **(D)** Scatter plot of correlation between pyroptosis risk score and tumor mutational burden. **(E)** Dot plot showing the pyroptosis risk score of 10 driver genes in gastric cancer regarding each mutation status.

To investigate the molecular characteristics of the different tiers of pyroptosis risk score, we analyzed the relationship among pyroptosis cluster, pyroptosis risk score and survival status in the TCGA cohort (Figure 3A). Patients in Cluster 3 had the lowest pyroptosis risk score compared with those in Cluster 1 and Cluster 2 (*p* < 0.001; Figure 3B). The distribution of pyroptosis risk score in the TCGA molecular subtypes was also investigated, and the MSI subtype was found to have the lowest pyroptosis risk score, followed by the EBV subtype (*p* < 0.001; Figure 3C). Interestingly, pyroptosis risk score was negatively associated with TMB (r = −0.39, *p* < 0.001; Figure 3D). We further explored the impact of mutational status of 10 common driver genes on pyroptosis risk score in GC (Wang et al., 2011), and found that pyroptosis risk scores increased in patients with *TP53* mutation, but decreased in patients with mutations in *ARID1A*, *PIK3CA*, *KMT2D*, *DNAH7*, *TTN*, *ACVR2A*, *CTNNB1*, *RPL21*, or *FMN2* (all *p* < 0.05; Figure 3E).

### Prognostic Performance of Pyroptosis Risk Score

**FIGURE 4:**
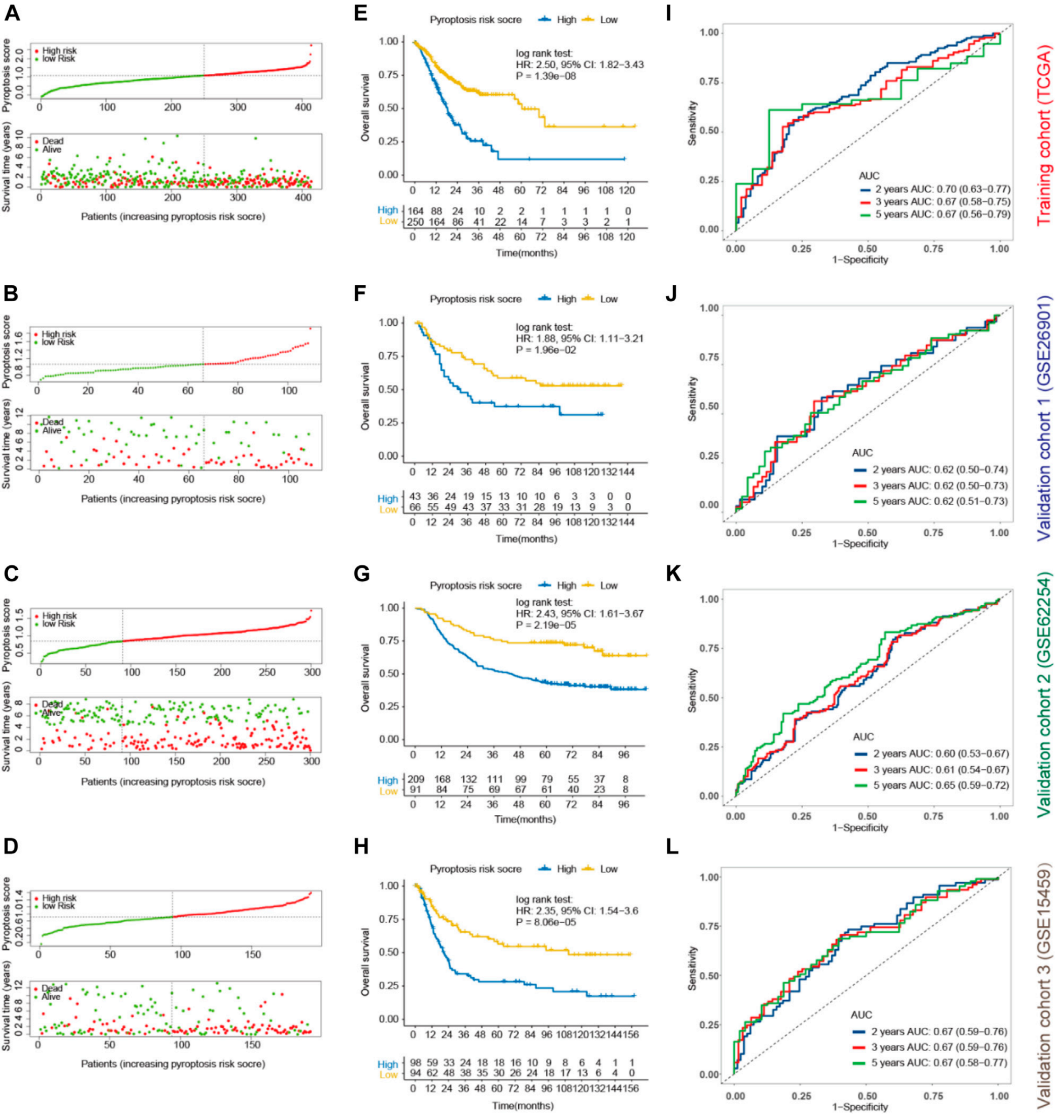
Prognostic values of pyroptosis risk score for OS in gastric cancer. Patients were divided into high- and low-pyroptosis risk score groups according to the optimal cutoff value using “survminer” package in the training (TCGA; **(A)**, validation cohort-1 (GSE26901; **(B)**, validation cohort- 2 (GSE62254; **(C)** and validation cohort-3 (GSE15459; **(D)**. Kaplan-Meier curves of OS of the high- and low-pyroptosis risk score groups in the training **(E)**, validation cohort-1 **(F)**, validation cohort-2 **(G)** and validation cohort-3 **(H)**. Time-dependent ROC for 2, 3, 5 years based on pyroptosis risk score in the training **(I)**, validation cohort-1 **(J)**, validation cohort-2 **(K)** and validation cohort-3 **(L)** cohorts. OS, overall survival; ROC, receiver operating characteristics curves.

To validate the prognostic potential of pyroptosis risk score in GC, three external cohorts (GSE26901 (Oh et al., 2018), GSE62254 (Cristescu et al., 2015), and GSE15459 (Ooi et al., 2009)) with available mRNA expression data of the 8 genes identified by the LASSO model were included in our study. Supplementary Table S2 shows the baseline co-variates of the TCGA and the three GEO cohorts. Patients were divided into high- and low-pyroptosis risk score groups according to the optimal cutoff value as determined in each cohort (Figures 4A–E). Kaplan-Meier curves revealed that shorter OS was observed in subjects with high-pyroptosis risk score in the training cohort (HR, 2.50; *p* < 0.001; Figure 4E), validation cohort-1 (HR, 1.88; *p* = 0.020; Figure 4F), validation cohort-2 (HR, 2.43; *p* < 0.001; Figure 4G), and validation cohort-3 (HR, 2.35; *p* < 0.001; Figure 4H), compared with those with low-pyroptosis risk score.

Furthermore, time-dependent ROC analysis was performed to evaluate the prognostic accuracy of pyroptosis risk score in the training and validation cohorts. As most of the patients did not reach the end point for 1-year, we analyzed the expression index of AUCs for 2-year instead. The AUC values of the pyroptosis risk score for 2-, 3- and 5-year OS were 0.70 (95% CI, 0.63–0.77), 0.67 (0.58–0.75) and 0.67 (0.56–0.79) in the training cohort, respectively (Figure 4I). Similar results were observed in the validation cohorts, with the respective AUCs of 2-, 3-, and 5-year OS as 0.62 (0.50–0.74), 0.62 (0.50–0.73) and 0.62 (0.51–0.73) in the validation cohort-1 (Figure 4J), 0.60 (0.53–0.67), 0.61 (0.54–0.67) and 0.65 (0.59–0.72) in the validation cohort-2 (Figure 4K), and 0.67 (0.59–0.76), 0.67 (0.59–0.76) and 0.67 (0.58–0.77) in the validation cohort-3 (Figure 4L). Multivariable Cox regression analysis further demonstrated that the pyroptosis risk score served as an independent predictor of OS in the training and validation cohorts, with HRs of 2.43 (1.74–3.40; *p* < 0.001), 1.83 (1.05–3.22; *p* = 0.034), 1.78 (1.16–3.72; *p* = 0.008) and 2.35 (1.54–3.60; *p* < 0.001), respectively (Table 1). Parallel results of RFS were observed in the validation cohort-1 and 2 (Supplementary Figure S4).

**TABLE 1:** Uni- and multivariable Cox regression in the training and validation cohorts.

	Univariable		Multivariable	
	HR (95% CI)	*p*	HR (95% CI)	*p*
Training cohort (TCGA)				
Pyroptosis risk score (high vs. low)	2.50 (1.82–3.43)	**<0.001**	2.43 (1.74–3.40)	**<0.001**
Gender (male vs. female)	1.22 (0.87–1.71)	0.240	—	—
Age (>60 vs. ≤ 60 years)	1.55 (1.09–2.21)	**0.015**	2.06 (1.41–3.02)	**<0.001**
Location (non-EGJ vs. EGJ)	0.93 (0.64–1.37)	0.726	—	—
Grade (G3 vs. G1/G2)	1.45 (1.04–2.02)	0.030	1.28 (0.90–1.82)	0.171
AJCC stage (III/IV vs. I/II)	1.98 (1.41–2.78)	**<0.001**	1.91 (1.35–2.70)	**<0.001**
MSI Status (MSI vs. MSS/MSI-L)	0.70 (0.45–1.09)	0.112	—	—
Validation cohort 1 (GSE26901)				
Pyroptosis risk score (high vs. low)	1.88 (1.11–3.21)	**0.020**	1.83 (1.05–3.22)	**0.034**
Gender (male vs. female)	1.39 (0.78–2.47)	0.259	—	—
Age (>60 vs. ≤ 60 years)	2.90 (1.70–4.94)	**<0.001**	2.69 (1.53–4.71)	**0.001**
Location (non-EGJ vs. EGJ)	0.88 (0.43–1.81)	0.736	—	—
Lauren (Diffuse vs. Intestinal/mixed)	0.92 (0.39–2.14)	0.839	—	—
AJCC stage (III/IV vs. I/II)	4.29 (2.38–7.72)	**<0.001**	3.23 (1.75–5.96)	**<0.001**
Validation cohort 2 (GSE62254)				
Pyroptosis risk score (high vs. low)	2.43 (1.61–3.67)	**<0.001**	1.78 (1.16–2.72)	**0.008**
Gender (male vs. female)	0.90 (0.65–1.27)	0.559	—	—
Age (>60 vs. ≤ 60 years)	1.26 (0.91–1.76)	0.168	—	—
Location (non-EGJ vs. EGJ)	0.58 (0.38–0.89)	**0.013**	0.74 (0.48–1.14)	0.172
Lauren (Diffuse vs. Intestinal/mixed)	1.52 (1.10–2.09)	**0.010**	1.07 (0.77–1.50)	0.672
AJCC stage (III/IV vs. I/II)	3.41 (2.34–4.96)	**<0.001**	2.94(1.98–4.36)	**<0.001**
Validation cohort 3 (GSE15459)				
Pyroptosis risk score (high vs. low)	2.35 (1.54–3.60)	**<0.001**	2.35 (1.54–3.60)	**<0.001**
Gender (male vs. female)	1.40 (0.91–2.17)	0.127	—	—
Age (>60 vs. ≤ 60 years)	0.98 (0.64–1.51)	0.936	—	—
Lauren (Diffuse vs. Intestinal/mixed)	1.19 (0.79–1.79)	0.401	—	—

AJCC, American Joint Committee on Cancer; EGJ, esophagogastric junction; MSI-H, microsatellite instability-high; MSI-L, microsatellite instability-low; MSS, microsatellite instability stable. Bold values is meaning these value less than 0.05.

**FIGURE 5:**
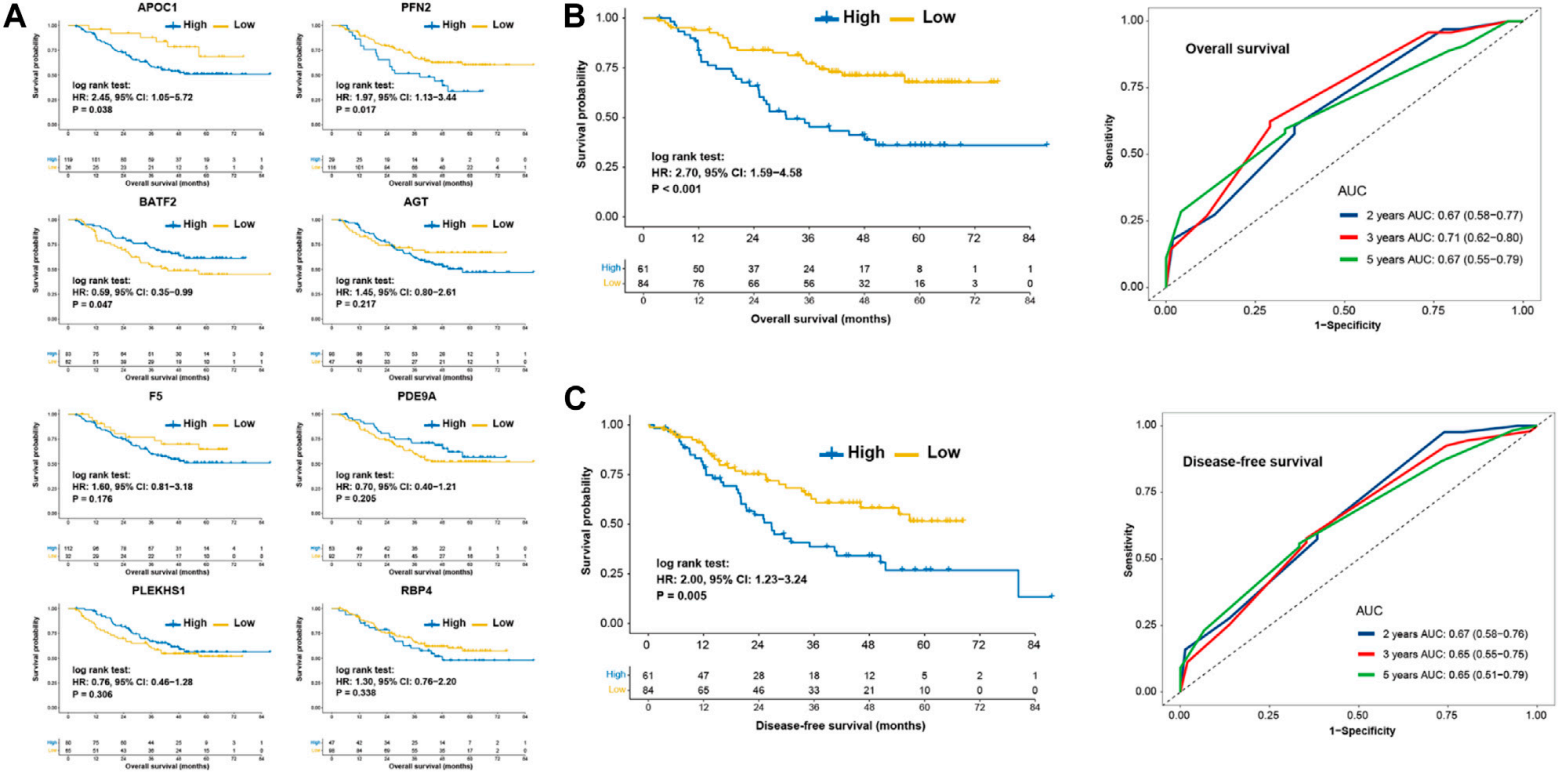
Prognostic values of pyroptosis risk score in the SYSUCC cohort. **(A)** Kaplan-Meier curves of overall survival of patients with low- or high-expression of 8 eight genes. Kaplan-Meier curve and Time-dependent ROC for 2, 3, 5 years based on pyroptosis risk score in the SYSUCC cohort regarding overall survival. **(B)** and disease-free survival. **(C)** SYSUCC, Sun Yat-Sen University Cancer center.

To further investigate the prognostic value of pyroptosis risk score, quantitative real-time RT-PCR from the real-world validation cohort of Sun Yat-Sen University Cancer center (SYSUCC) were performed. A total of 145 patients of SYSUCC were included. Survival analysis showed that patients with high expression of APOC1 (HR, 2.45; *p* = 0.038) or PFN2 (HR, 1.97; *p* = 0.017) was associated with shorter OS, and patients with high expression of BATF2 (HR, 0.59; *p* = 0.047) was associated with prolonged OS (Figure 5A). The other five genes were not associated with the survival (Figure 5B). Parallel results of DFS were observed in the SYSUCC cohort (Figure 5C). Multivariable Cox regression analysis further demonstrated that the pyroptosis risk score served as an independent predictor of OS and DFS in the SYSUCC cohort, with HRs of 2.67 (1.53–4.65; *p* < 0.001) and 1.72 (1.05–2.83; *p* = 0.033), respectively (Table 2).

**TABLE 2:** Uni- and multivariable Cox regression of OS and DFS in the SYSUCC cohort.

	Univariable		Multivariable	
	HR (95% CI)	*P*	HR (95% CI)	*p*
OS				
Pyroptosis risk score (high vs. low)	2.70 (1.59–4.58)	**<0.001**	2.67 (1.53–4.65)	**<0.001**
Gender (male vs. female)	1.38 (0.77–2.50)	0.275	—	—
Age (>60 vs. ≤ 60 years)	1.88 (1.08–3.25)	**0.024**	1.56 (0.86–2.83)	0.142
Location (non-EGJ vs. EGJ)	1.22 (0.72–2.05)	0.461	—	—
Grade (G3 vs. G1/G2)	0.96 (0.57–1.61)	0.880	—	—
Lauren (Diffuse vs. Intestinal/mixed)	1.02 (0.60–1.71)	0.951	—	—
Nerve invasion (yes vs. no)	1.38 (0.68–2.82)	0.372	—	—
Vascular invasion (yes vs. no)	1.67 (0.97–2.88)	0.062	—	—
Tumor size (>5 vs. ≤ 5 cm)	3.24 (1.68–6.26)	**< 0.001**	2.57 (1.28–5.16)	**0.008**
CEA (>5 vs. ≤ 5 ng/ml)	1.85 (1.08–3.16)	**0.025**	1.56 (0.87–2.80)	0.134
CA19-9 (>35 vs. ≤ 35 U/mL)	1.07 (0.15–7.89)	0.947	—	—
CA72-4 (>5 vs. ≤ 5 U/mL)	1.12 (0.23–3.03)	0.828	—	—
AJCC stage (III vs. II)	3.89 (1.67–9.07)	**0.002**	2.86 (1.19–6.82)	**0.018**
Adjuvant chemotherapy (yes vs. no)	0.38 (0.23–0.64)	**< 0.001**	0.45 (0.26–0.80)	**0.006**
DFS				
Pyroptosis risk score (high vs. low)	2.00 (1.23–3.24)	**0.005**	1.72 (1.05–2.83)	**0.033**
Gender (male vs. female)	1.66 (0.96–2.86)	0.068	—	—
Age (>60 vs. ≤ 60 years)	1.56 (0.95–2.56)	0.077	—	—
Location (non-EGJ vs. EGJ)	1.12 (0.69–1.83)	0.640	—	—
Grade (G3 vs. G1/G2)	1.01 (0.63–1.64)	0.955	—	—
Lauren (Diffuse vs. Intestinal/mixed)	1.03 (0.64–1.65)	0.915	—	—
Nerve invasion (yes vs. no)	1.60 (0.82–3.14)	0.171	—	—
Vascular invasion (yes vs. no)	1.83 (1.10–3.03)	**0.019**	1.37 (0.81–2.32)	0.241
Tumor size (>5 vs. ≤ 5 cm)	3.53 (1.89–6.60)	**< 0.001**	3.09 (1.59–5.98)	**0.001**
CEA (>5 vs. ≤ 5 ng/ml)	1.59 (0.95–2.64)	0.076	—	—
CA19-9 (>35 vs. ≤ 35 U/ml)	1.07 (0.15–7.89)	0.947	—	—
CA72-4 (>5 vs. ≤ 5 U/ml)	1.27 (0.17–9.32)	0.966	—	—
AJCC stage (III vs. II)	2.99 (1.48–6.06)	**0.002**	1.99 (0.97–4.08)	0.061
Adjuvant chemotherapy (yes vs. no)	0.53 (0.33–0.86)	**0.011**	0.63 (0.38–1.05)	0.076

OS, overall survival; DFS, disease-free survival; SYSUCC, Sun Yat-Sen University Cancer center; AJCC, American Joint Committee on Cancer; EGJ, esophagogastric junction; CEA, carcinoma embryonic antigen; CA19-9, carbohydrate antigens 19-9; CA72-4, carbohydrate antigens 72-4. Bold values is meaning these value less than 0.05.

**FIGURE 6:**
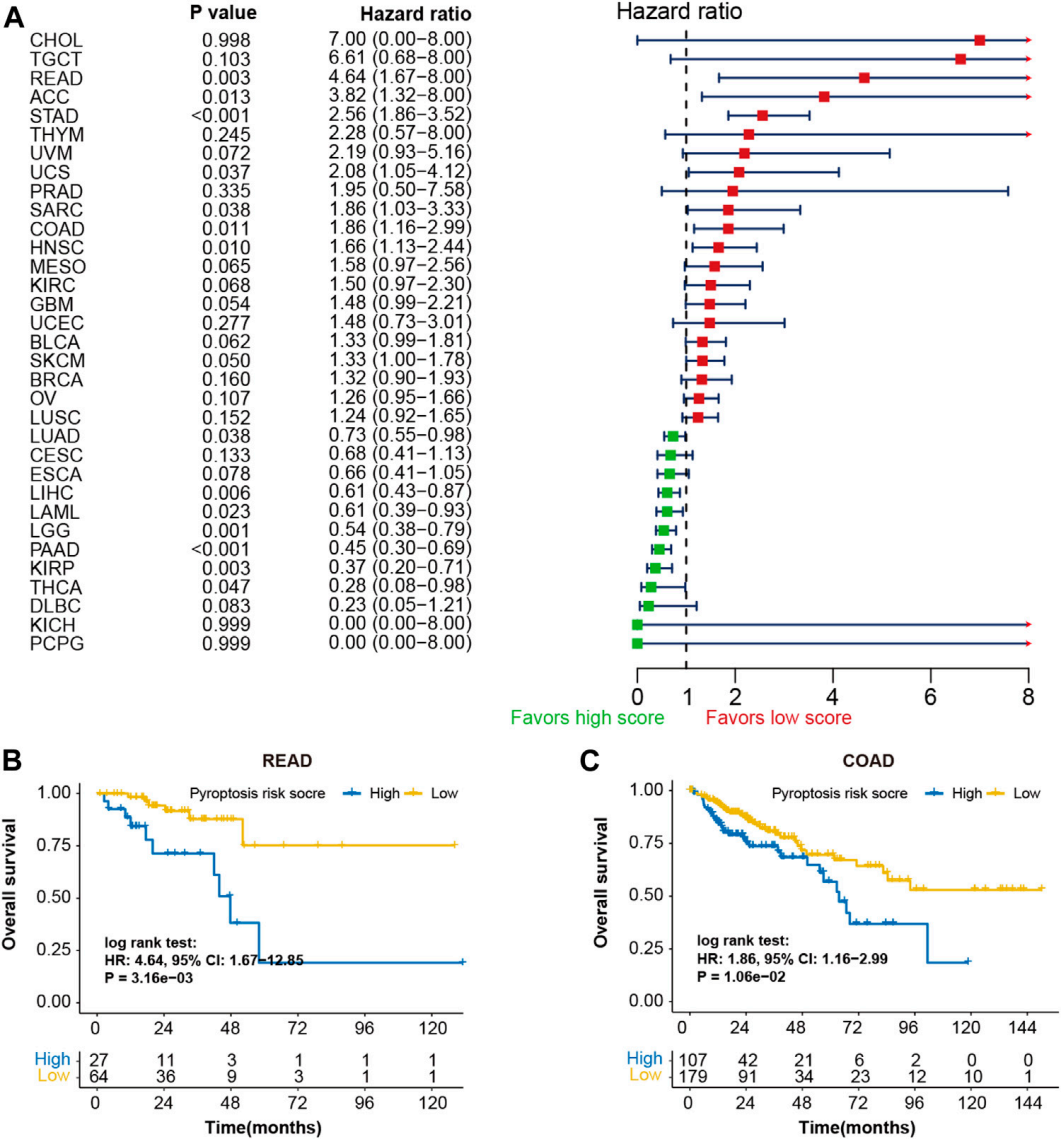
Prognostic values of pyroptosis risk score in 33 cancer types. **(A)** Forest plot depicting the hazard ratios for pyroptosis risk score in 33 cancer types of the TCGA cohorts. Kaplan-Meier curves of overall survival of patients with low- or high-pyroptosis risk score in rectum adenocarcinoma **(B)** and colon adenocarcinoma **(C)**.

The prognostic potentials of pyroptosis risk score in 33 cancer types were further investigated in the Pan-Cancer TCGA cohort (Figure 6A). Patients with high-pyroptosis risk score exhibited shorter survival in rectum carcinoma (Figure 6B), adrenocortical carcinoma, uterine carcinosarcoma, sarcoma, colon carcinoma (Figure 6C), head and neck squamous cell carcinoma, and skin cutaneous melanoma. However, prolonged survival was observed in cervical squamous cell carcinoma and endocervical adenocarcinoma, liver cancer, acute myeloid leukemia, brain lower grade glioma, pancreatic cancer, kidney renal clear cell carcinoma, and thyroid carcinoma.

### Construction of Merged Score to Outstrip the Prediction of TNM Stage

**FIGURE 7:**
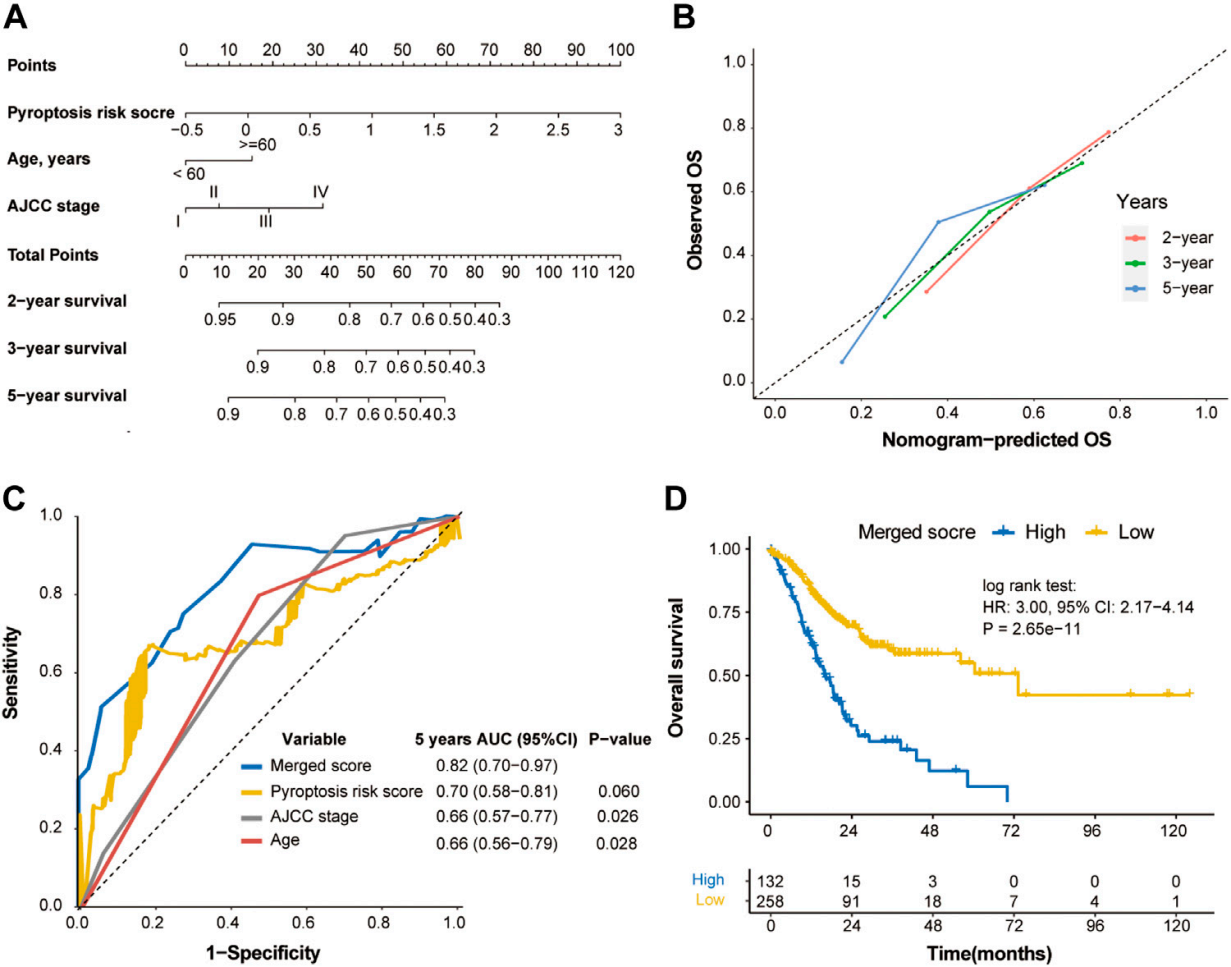
Construction of merged risk score in the TCGA cohort. **(A)** A merged score was constructed by nomogram COX regression based on the pyroptosis risk score, age and TNM stage in the TCGA cohort. **(B)** Calibration curve of the nomogram regarding the predicted and observed survival. **(C)** Comparison of the merged score and other variables. **(D)** Kaplan-Meier curves of OS of high- and low-merged score. OS, overall survival.

Based on the multivariable Cox regression analysis on the training cohort, pyroptosis risk score, age, and TNM stage were used to generate a merged score to better predict the probability of OS through a nomogram model (Figure 7A). The calibration curve indicated excellent goodness of fit between predicted and observed 2-, 3- and 5-year OS (Figure 7B). The merged score had significant higher AUC [0.82 (0.70–0.97)] than TNM stage (*p* = 0.026; Figure 7C), and demonstrated great prognostic performance (HR, 3.00; *p* < 0.001; Figure 7D).

**FIGURE 8:**
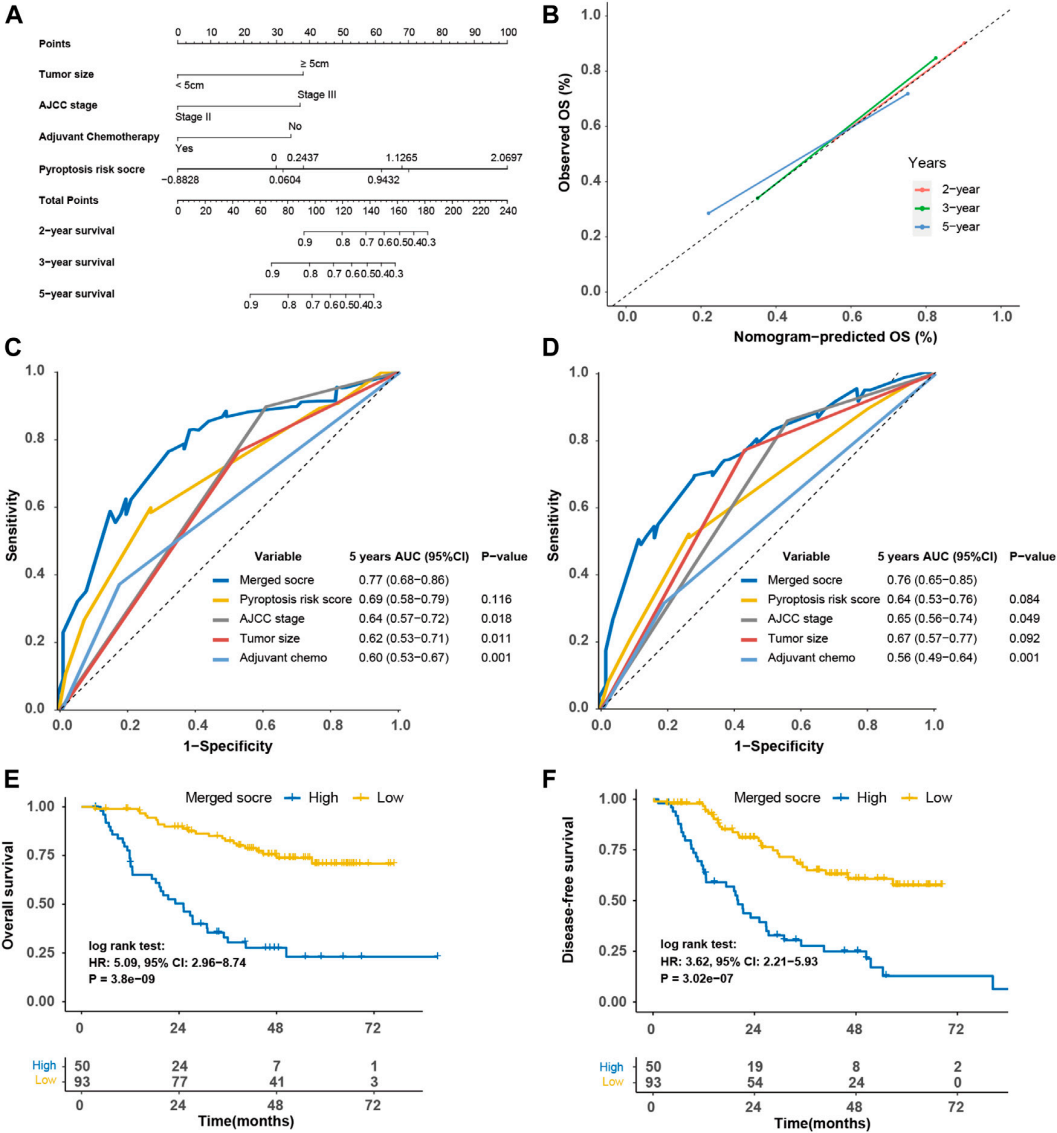
Construction of merged risk score in the SYSUCC cohort. **(A)** A merged score was constructed by nomogram COX regression based on the pyroptosis risk score, tumor size, adjuvant chemotherapy and TNM stage in the SYSUCC cohort. **(B)** Calibration curve of the nomogram regarding the predicted and observed survival. Comparison of the merged score and other variables in term of overall survival **(C)** and disease-free survival **(D)**. Kaplan-Meier curves of overall survival **(E)** and disease-free survival **(F)** of high- and low-merged score. SYSUCC, Sun Yat-Sen University Cancer center.

The nomogram based on the tumor size, TNM stage, adjuvant chemotherapy, and pyroptosis risk score in the SYSUCC cohort was generated (Figures 8A,B). Similarly, the merged score also had higher AUC of OS [0.77 (0.68–0.86)] and DFS [0.76 (0.53–0.74)] than TNM stage (*p* = 0.018, Figure 8C; *p* = 0.049, Figure 8D). The HRs of the merged score for OS and DFS were 5.09 (2.96–8.74) (Figure 8E) and 3.62 (2.21–5.93) (Figure 8F) in the SYSUCC cohort, respectively.

### Regulation and Biological Phenotypes of Pyroptosis Risk Score

**FIGURE 9:**
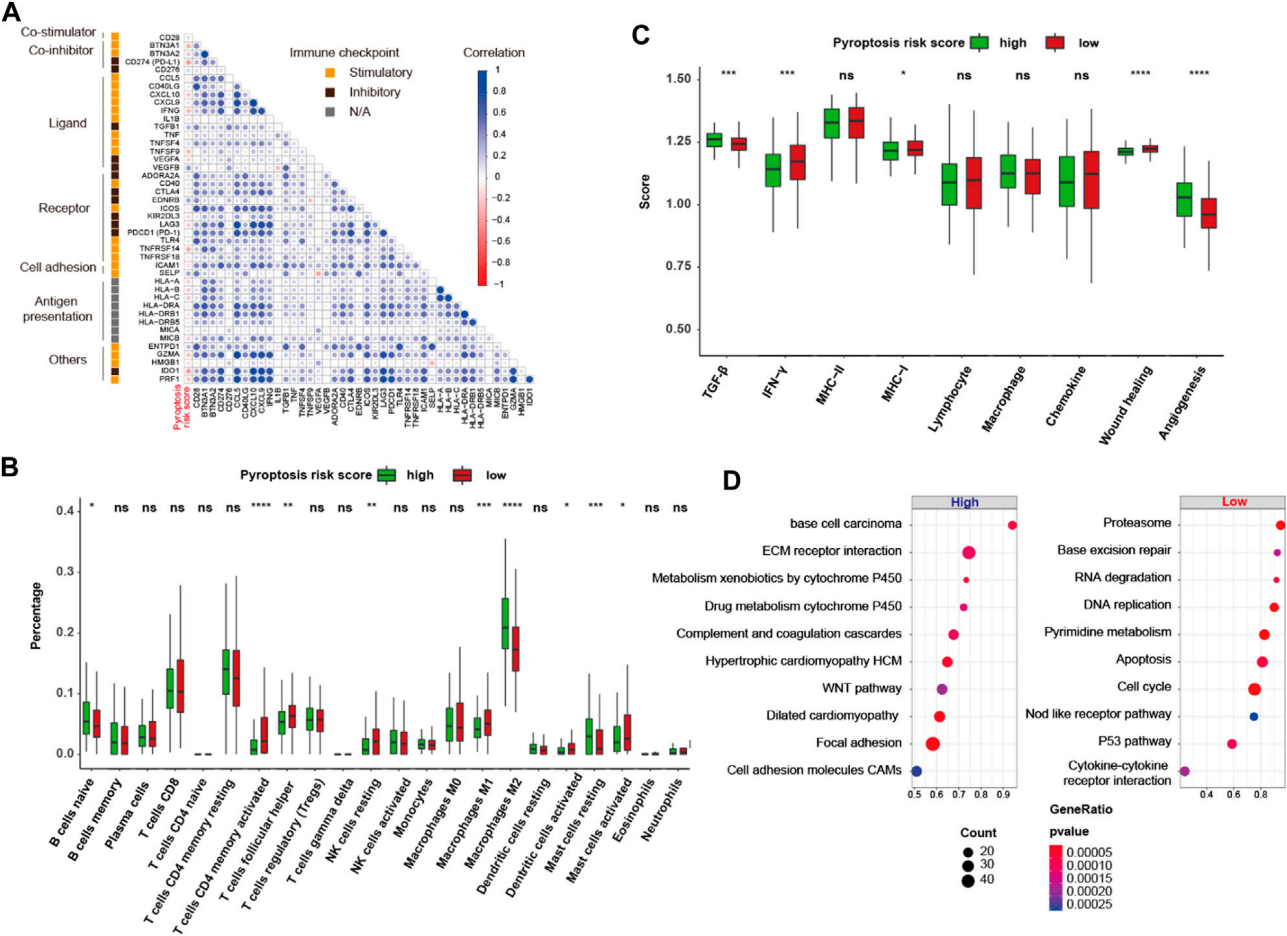
Gene regulation and biological phenotypes of the high- and low-pyroptosis risk score groups. **(A)** Correlation matrix heatmap of stimulatory and inhibitory immune checkpoints. **(B,C)** Proportion of the 22 immune cell fractions and immune signatures between the high- and low-pyroptosis risk score groups. **(D)** Bubble plot of top 10 enrichment GSEA pathways. ns: not significant; *p < 0.05; **p < 0.01; ***p < 0.001; ****p < 0.0001.

To further interrogate its potential, the regulation and biological phenotypes associated with pyroptosis risk score in TIME are needed. First, the correlation between pyroptosis risk score and mRNA expression of immunomodulators (IMs) was derived. Figure 9A depicts a significant correlation between pyroptosis risk score and gene expression of IMs (all *p* < 0.05). Of note, pyroptosis risk score reversely correlated with expression of *PD-L1*, *LAG3*, *IDO1*, and other antigen presentation genes (all *p* < 0.05). In addition, significantly higher proportions of activated CD4 memory T cells, T follicular helper cells and M1 macrophage, and lower proportions of M2 macrophage were observed in the low-pyroptosis risk score group (Figure 9B). Immune signature analysis also indicated that the low-pyroptosis risk score group had higher scores of IFN-γ, MHC-I and wound healing, but lower scores of TGF-β and angiogenesis (Figure 9C). GSEA analysis revealed that the pathways significantly enriched in the low-pyroptosis risk score group were mainly related to DNA damage repair (DDR), such as base excision repair, DNA replication, and P53 pathways (Figure 9D). Other enriched pathways related to Nod-like receptors (NLRs), apoptosis, and cell cycle were also observed, which further validated the relatively high levels of pyroptosis in the low-pyroptosis risk score group.

### Prediction of Response to Immunotherapy by Pyroptosis Risk Score

**FIGURE 10:**
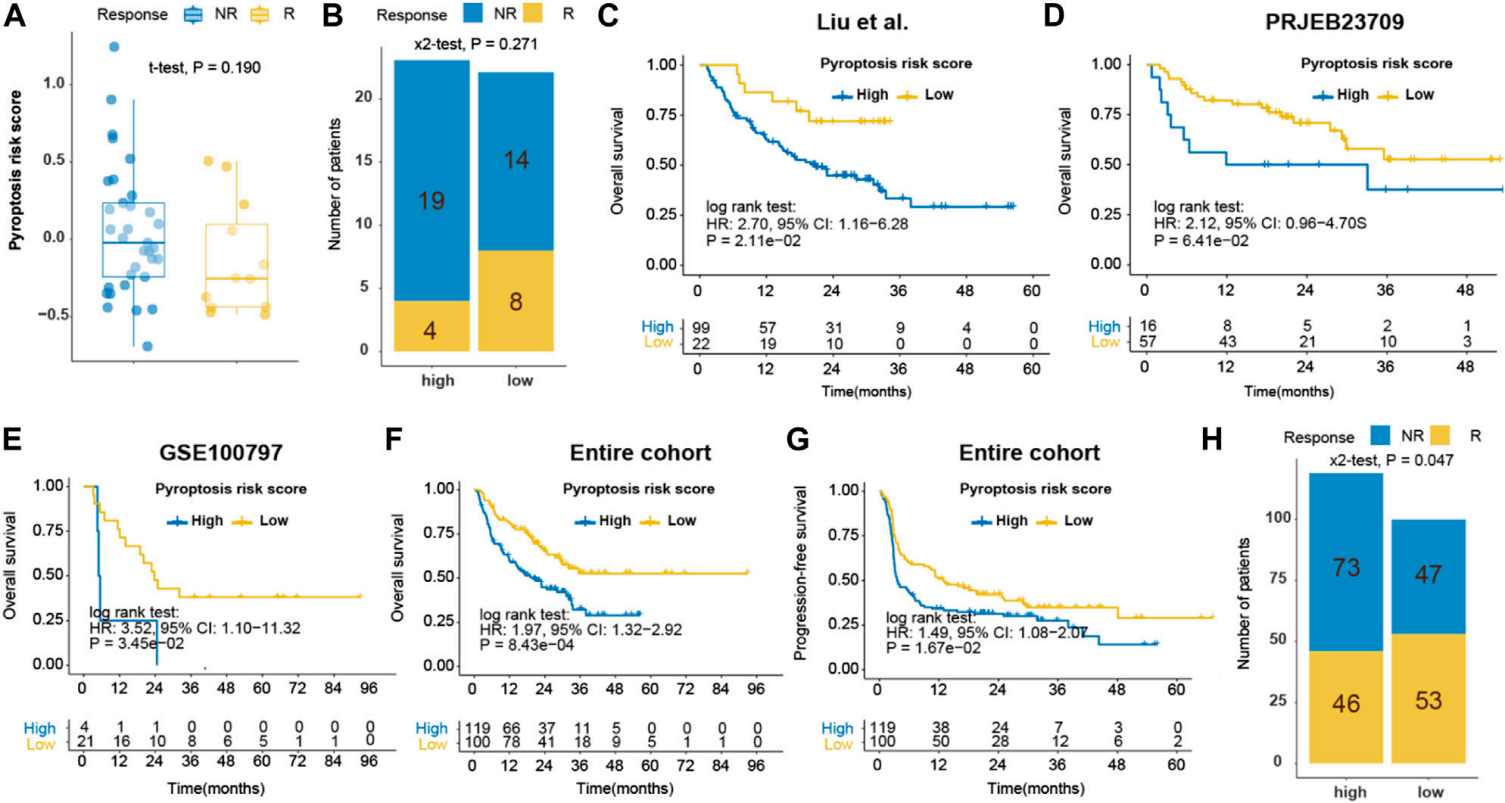
Prediction of immune response by pyroptosis risk score in ICB cohorts. **(A)** Dot plot of pyroptosis risk score for responder and non-responder to ICB in the Kim cohort (gastric cancer); **(B)** Bar plot of pyroptosis risk score and response to ICB in the Kim cohort; Kaplan-Meier curves of OS of patients with high- or low-pyroptosis risk score after ICB in the Liu **(C)**, PRJEB23709 **(D)**, GSE100797 **(E)** and entire **(F)** melanoma cohorts. **(G)** Kaplan-Meier curves of PFS of patients with low- and high-pyroptosis risk score after ICB in the entire melanoma cohorts. **(H)** Bar plot of pyroptosis risk score and response to ICB in the entire melanoma cohort. ICB, immune checkpoint blockade. OS, overall survival. PFS, progression-free survival.

The inflamed nature and enriched DDR pathways in the low-pyroptosis risk score group implied the possibility of using pyroptosis risk score as a predictor of response to ICB. In the ICB cohort of metastatic GC (*n* = 45), responders exhibited a tendency of having lower pyroptosis risk score (−0.135 ± 0.363 vs. 0.049 ± 0.422, *p* = 0.190, power for test: 0.280; Figure 10A), compared with non-responders. The objective response rate (ORR) of patients with low- and high-pyroptosis risk score was 36.4% (8/22) versus 17.4% (4/23) (*p* = 0.271, power for test: 0.170; Figure 10B). In addition, the practicability of pyroptosis risk score was investigated in the context of immunotherapy in the melanoma cohorts. Patients with high-pyroptosis risk score exhibited significantly or marginally significantly shorter OS in the Liu (HR, 2.70; *p* = 0.021; Figure 10C), PRJEB23709 (HR, 2.12; *p* = 0.064; Figure 10D) and GSE100797 (HR, 3.52; *p* = 0.035; Figure 10E) cohorts, compared with those with low score. Analysis of the entire cohort further demonstrated shorter OS (HR, 1.97; *p* < 0.001; Figure 10F) and PFS (HR, 1.49; *p* = 0.017; Figure 10G) in high-pyroptosis risk score group. The ORR of patients with low- and high-pyroptosis risk score was 53.0% (53/100) versus 38.7% (46/119) (*p* = 0.047; Figure 10H). Collectively, these findings indicated that pyroptosis risk score may serve as a predictor for ICB.

### Potential Antitumor Drugs Screening Based on Pyroptosis Risk Score

**FIGURE 11:**
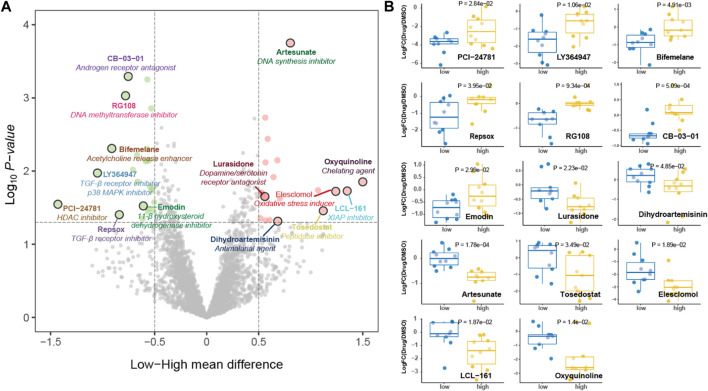
Potential antitumor drug screening based on pyroptosis risk score. **(A)** Volcano plot depicting the sensitive drugs for gastric cancer cell lines with high- (red) and low- (green) pyroptosis risk score. **(B)** Dot plot showing the fold change of viability values treated with potential targeted drugs compared with DMSO as transformed by log2.

Given the limited options of antitumor drugs used in clinical practice for GC, we conducted screening of potential antitumor drugs based on pyroptosis risk score using the PRISM drug repurposing resource. The potential sensitive drugs for GC cell lines are shown in Figure 11A. Of note, PCI-24781 [histone deacetylase (HDAC) inhibitor], LY364947 (TGF-β receptor inhibitor), bifemelane (acetylcholine release enhancer), repsox (TGF-β receptor inhibitor), and RG108 (DNA methyltransferase inhibitor) are the sensitive drugs for cell lines with a low-pyroptosis risk score, while oxyquinoline (chelating agent), LCL-161 (XIAP inhibitor) and artesunate (DNA synthesis inhibitor) are the sensitive drugs for cell lines with high-pyroptosis risk score (Figure 11B).

## Discussion

This is the first study to comprehensively analyze the pyroptosis pattern and its association with TIME features in GC. In the current study, a pyroptosis risk scoring system was proposed to quantify the risk for pyroptosis and to predict the survival of individuals and their response to immunotherapy. In addition, pyroptosis risk score was used to screen potential antitumor drugs for GC, which demonstrated its potential in assisting oncologists with making more efficient and personalized therapeutic decisions.

Pyroptosis is a GSDM-mediated inflammatory cell death activated by invasive infection and danger signals (Zheng et al., 2021). Previous studies showed that different GSDMs may play a reversing role in the tumorigenesis of GC (Saeki et al., 2009; Komiyama et al., 2010; Qiu et al., 2017; Wang et al., 2018). Although GSDMB was overexpressed in GC and might act as an oncogene (Saeki et al., 2009), GSDMD was found to be downregulated in GC and could protect against the proliferation of GC through inhibiting S/G2 transition and the STAT3/PI3K/PK8 pathways (Wang et al., 2018). These findings suggest that the role and pattern of pyroptosis in GC deserve further exploration. Based on the expression profiles of pyroptosis genes, three distinct molecular clusters were identified for GC. Among these, Cluster 3 was positively correlated with the expression of *CASP1*, *CASP4*, *CASP5*, *GSDMD*, *IL1B* and *IL18*, indicating that Cluster 3, to some extent, represented the high levels of pyroptosis in the canonical or non-canonical pathway. Exploration of immunogenetic characteristics, genomic features, and prognosis among the pyroptosis clusters further suggested that Cluster 3 were inflamed tumors, therefore, showing the most promising prognosis.

Given the subpar prognostic performance of the TNM staging system, pyroptosis risk scoring system was constructed to predict the risk of pyroptosis and the survival of GC patients. The robustness and reproducibility of pyroptosis risk scoring system in the TCGA and three validation cohorts suggest that pyroptosis risk score is a powerful tool for predicting the clinical outcomes of patients with GC. To validate these analyses by in silico algorithm, quantitative real-time RT-PCR from the real-world validation cohort from SYSUCC cohort were performed. The prognostic value of pyroptosis risk scoring system were validated in the SYSUCC cohort. Previous studies reported that tumor-associated M2 macrophages could promote the metastasis of GC via the secretion of growth factors (Chen et al., 2017), which could be used to predict GC survival and chemotherapy benefit (Jiang et al., 2018). In our study, dominant M1 macrophages and a decrease in M2 macrophages were observed in the low-pyroptosis risk score group. Likewise, higher fractions of activated CD4 memory T cells and T follicular helper cells were noted in the same group. Therefore, an activated immune environment in subjects with low-pyroptosis risk score may help explain their better prognosis. Consistent with previous studies, we found that pyroptosis risk score could serve as either a favorable or harmful factor in different cancer types, indicating that pyroptosis could act as a double-edged sword in malignant tumor (Yu et al., 2021).

Despite the clinical benefit of anti-PD-1 therapy across a wide range of malignancies (Ribas and Wolchok, 2018), only a small subset of GC patients exhibited durable response (Boku et al., 2021; Janjigian et al., 2021). Therefore, a reliable predictor that can forecast patients’ response to ICB is urgently needed. Since the reinvigoration of pyroptosis can lead to the release of inflammatory cytokines, such as IL-1β and IL-18, pyroptosis can theoretically perform crosstalk with antitumor immunity (Ren et al., 2020), and may even predict response to ICB. However, very little is known about the crosstalk between pyroptosis and antitumor immunity in GC. It is recognized that patients with EBV positivity derive greater clinical benefit from ICB in the context of GC (Kim et al., 2018), so are those with MSI-high status (Le et al., 2015; Fuchs et al., 2018; Kim et al., 2018) and high TMB (Yang et al., 2020). In our study, we found that a lower pyroptosis risk score was detected in the MSI-high subtype. Moreover, higher TMB, PD-L1 expression, antigen presentation, IFN-γ signature and enriched DDR pathways were also found in patients with low-pyroptosis risk score, which suggested the potential predictive value of pyroptosis risk scoring system with regard to response to ICB. Consistently, low-pyroptosis risk score was also found to be associated with improved response and survival in subjects with GC (did not reach statistic difference because of small sample size) and melanoma. Therefore, pyroptosis risk scoring system may be helpful in the selection of GC patients who may clinically benefit from ICB.

GC is only sensitive to a limited amount of cytotoxic agents and HER2 antibody (HER2-positive) (Bang et al., 2010; Shitara et al., 2020b), and more novel and effective antitumor drugs should be explored. Using the PRISM drug repurposing resource (Corsello et al., 2020), we evaluated over 4000 drugs for GC. Specifically, we identified that cell lines with low-pyroptosis risk score were more sensitive to the inhibitors of HDAC and TGF-β receptor. It has been reported that HDAC inhibitors can alter the subgroup of CD4 and CD8 tumor-infiltrating T cells in colorectal cancer (Blaszczak et al., 2021) and enhance the effectiveness of immunotherapy in multiple myeloma (Hirano et al., 2021). Compared with PD-1 monotherapy, selective blocking of PD-1 and TGF-β pathways can enhance antitumor activity (Lan et al., 2018; de Streel et al., 2020). Therefore, the antitumor activity of HDAC or TGF-β inhibitors, combining with PD-1 antibody, in GC may warrant further investigation *in vitro* and *in vivo*.

Despite providing a more comprehensive prospective on pyroptosis in GC, our study has several limitations. Currently, the cornerstone drugs for GC are cytotoxic. However, the predictive ability of pyroptosis risk scoring system in chemotherapy benefit could not be assessed as the eligible cohort (GSE26901) that includes adjuvant chemotherapy data did not show chemotherapy benefit (HR, 1.51; *p* = 0.100) for GC. The real-world validation of SYSUCC cohort validate the survival benefit of adjuvant chemotherapy. However, the pyroptosis risk scoring system could not predict the chemotherapy benefit in the real-world validation cohort (Supplementary Figure S5). In addition, distinct pyroptosis and TIME features in different tumor regions, such as intratumoral and peritumoral regions, could not be evaluated from bulk tissue RNA profiles. Lastly, this study is naturally retrospective, and confirmation of our results in a prospective clinical trial is needed.

In conclusion, our study provides crucial clues for the crosstalk between pyroptosis and TIME features in GC. The pyroptosis-based risk score can serve as an independent predictor for individual survival and response to immunotherapy. This score may also assist in the screening of potential antitumor drugs for GC.

## Data Availability

The original contributions presented in the study are included in the article/Supplementary Material; further inquiries can be directed to the corresponding authors. Our RDD number is RDDA2022792096 (www.researchdata.org.cn).
