# Supplementary material for: Genomic Analysis Uncovers the Prognostic and Immunogenetic Feature of Pyroptosis in Gastric Carcinoma: Indication for Immunotherapy
Source: Front Cell Dev Biol. 2022 Jul 13;10:906759. doi: 10.3389/fcell.2022.906759 (PMC9328384; doi:10.3389/fcell.2022.906759)
Supplement: Supplementary file 1 [file DataSheet1.docx]

**Supplement Table 1**. List of the pyroptosis-related genes

| **Genes** | **Full names** | **Assortment** |
| --- | --- | --- |
| CASP1 | Caspase 1 | Caspase |
| CASP3 | Caspase 3 | Caspase |
| CASP6 | Caspase 6 | Caspase |
| CASP8 | Caspase 8 | Caspase |
| CASP9 | Caspase 9 | Caspase |
| CASP4 | Caspase 4 | Caspase |
| CASP5 | Caspase 5 | Caspase |
| CHMP2A | charged multivesicular body protein 2A | CHMP family |
| CHMP2B | charged multivesicular body protein 2B | CHMP family |
| CHMP3 | charged multivesicular body protein 3 | CHMP family |
| CHMP4A | charged multivesicular body protein 4A | CHMP family |
| CHMP4B | charged multivesicular body protein 4B | CHMP family |
| CHMP4C | charged multivesicular body protein 4C | CHMP family |
| CHMP6 | charged multivesicular body protein 6 | CHMP family |
| CHMP7 | charged multivesicular body protein 7 | CHMP family |
| IL1A | Interleukin 1 alpha | cytokine |
| IL6 | Interleukin 6 | cytokine |
| IL18 | Interleukin 18 | cytokine |
| IL1B | Interleukin 1 beta | cytokine |
| TNF | Tumor necrosis factor | cytokine |
| STAT3 | Signal transducer and activator of transcription 3 | cytokine |
| CD274 | cluster of differentiation 274 (Programmed death-ligand 1) | cytokine |
| GSDMD | Gasdermin D | Gasdermin superfamily |
| GSDMC | Gasdermin C | Gasdermin superfamily |
| DFNA5 | Gasdermin E | Gasdermin superfamily |
| GSDMB | Gasdermin B | Gasdermin superfamily |
| GSDMA | Gasdermin A | Gasdermin superfamily |
| DFNB59 | Pejvakin | Gasdermin superfamily |
| GZMA | Granzyme A | Granzyme-mediated |
| GZMB | Granzyme B | Granzyme-mediated |
| MEFV | Mediterranean fever | inflammasomes-mediated |
| PYCARD | PYD And CARD Domain Containing | inflammasomes-mediated |
| AIM2 | absent in melanoma 2 | inflammasomes-mediated |
| NLRP1 | NLR family pyrin domain containing 1 | NLR Family |
| NLRP2 | NLR family pyrin domain containing 2 | NLR Family |
| NLRP6 | NLR family pyrin domain containing 6 | NLR Family |
| NLRP7 | NLR family pyrin domain containing 7 | NLR Family |
| NLRC4 | NLR family CARD domain-containing protein 4 | NLR Family |
| NAIP | NLR Family Apoptosis Inhibitory Protein | NLR Family |
| NLRP3 | NACHT, LRR, FIIND, CARD domain and PYD domains-containing protein 7 | NLR Family |
| TLR4 | Toll-like receptor 4 | others |
| PRKACA | Protein Kinase CAMP-Activated Catalytic Subunit Alpha | others |
| IRF2 | Interferon regulatory factor 2 | others |
| TP63 | Tumor protein p63 | others |
| P2RX7 | P2X purinoceptor 7 | others |
| IRF1 | Interferon regulatory factor 1 | others |
| TP53 | Tumor protein P53 | others |
| HMGB1 | High mobility group box 1 | others |
| NOD1 | Nucleotide-binding oligomerization domain-containing protein 1 | others |
| NOD2 | Nucleotide-binding oligomerization domain-containing protein 2 | others |
| ELANE | Neutrophil elastase | others |
| PLCG1 | phospholipase C gamma 1 | others |
| GPX4 | Glutathione peroxidase 4 | others |
| BAK1 | BCL2 Antagonist/Killer 1 | others |
| BAX | BCL2 Associated X, Apoptosis Regulator | others |
| CYCS | cytochrome complex | others |
| SCAF11 | SR-Related CTD Associated Factor 11 | others |

**Supplement Table 2. Baseline co-variates of training and validation cohorts**

|  | **Training cohort** | **Validation cohort 1** | **Validation cohort 2** | **Validation cohort 3** |
| --- | --- | --- | --- | --- |
|  | **TCGA (n=414)** | **GSE26901 (n=109)** | **GSE62254 (n=300)** | **GSE15459 (n=192)** |
| Age, years Mean (SD) | 65.60 (10.74) | 55.94 (10.62) | 61.94 (11.36) | 64.37 (13.23) |
| Gender (%) |  |  |  |  |
| Male | 268 (64.7) | 69 (63.3) | 199 (66.3) | 125 (65.1) |
| Female | 146 (35.3) | 40 (36.7) | 101 (33.7) | 67 (34.9) |
| AJCC stage (%) |  |  |  |  |
| I | 58 (14.0) | 40 (36.7) | 30 (10.0) | 31 (16.1) |
| II | 122 (29.5) | 18 (16.5) | 97 (32.3) | 29 (15.1) |
| III | 122 (29.5) | 36 (33.0) | 96 (32.0) | 72 (37.5) |
| IV | 122 (29.5) | 15 (13.8) | 77 (25.7) | 60 (31.2) |
| Lauren (%) |  |  |  |  |
| Diffuse | - | 11 (10.1) | 135 (45.0) | 75 (39.1) |
| Intestinal | - | 82 (75.2) | 146 (48.7) | 99 (51.6) |
| Mixed | - | 5 (4.6) | 19 (6.3) | 18 (9.4) |
| Location (%) |  |  |  |  |
| EGJ | 89 (21.5) | 17 (15.6) | 36 (12.0) | - |
| Non-EGJ | 279 (67.4) | 92 (84.4) | 263 (87.7) | - |
| MSI status (%) |  |  |  |  |
| MSI-H | 73 (17.6) |  | 81 (27.0) | - |
| MSS/MSI-L | 306 (73.9) |  | 219 (73.0) | - |
| Molecular subtype (%) |  |  |  |  |
| CIN | 221 (53.4) | - | - | - |
| EBV | 30 (7.2) | - | - | - |
| GS | 50 (12.1) | - | - | - |
| HM-SNV | 7 (1.7) | - | - | - |
| MSI | 73 (17.6) | - | - | - |
| ACRG subtype (%) |  |  |  |  |
| EMT | - | - | 46 (15.3) | - |
| MSI | - | - | 68 (22.7) | - |
| TP53 negative | - | - | 107 (35.7) | - |
| TP53 positive | - | - | 81 (27.0) | - |
| Survival status |  |  |  |  |
| Dead | 160 (38.6) | 55 (50.5) | 152 (50.7) | 95 (49.5) |
| Alive | 254 (61.4) | 54 (49.5) | 148 (49.3) | 97 (50.5) |
| Survival time, months (median, range) | 14.1 (0-124.0) | 47.0 (2.0-140.0) | 57.9 (1.0-105.7) | 19.0 (0-157.8) |

AJCC, American Joint Committee on Cancer; EGJ, esophagogastric junction; MSI-H, microsatellite instability-high; MSI-L, microsatellite instability-low; MSS, microsatellite instability stable; CIN, chromosomal instability; EBV, Epstein Barr virus; GS, genome stability; HM-SNV, hypermutated single nucleotide variants; ACRG, Asian Cancer Research Group; EMT, epithelial-mesenchymal transition

**Supplement Table 3. The primer sequences**

| **genes** | **Forward primer** | **Reverse primer** | **Product length** |
| --- | --- | --- | --- |
| APOC1 | CTGGTGGTGGTTCTGTCGAT | TCACTCTGTTTGATGCGGCT | 149 |
| PDE9A | CAGAAGCAGCAGGACCAACT | GTAGTTGTCGTGGACGCAGA | 277 |
| F5 | TCGCCTCTGGGCTAATAGGA | GGGGTCATCACGTTTCACCT | 191 |
| PFN2 | CAACGGTTTGACTCTTGGCG | TGCCGACAGCCACATTGTAT | 131 |
| AGT | GCTGCAAAACTTGACACCGA | GGATTGCCTGTAGCCTGTCA | 211 |
| RBP4 | AGACTACGACACGTATGCCG | TGCCGCTGCCTTACAATCTT | 141 |
| PLEKHS1 | AGACATCCCATGAGTCTGTGG | CGGGGATATTACCCTTTTGGTC | 168 |
| BATF2 | ATGGATTGTGCCTCCTGCTC | AGGAGGTTCAGCAACCACAG | 246 |
| GAPDH | GCACCGTCAAGGCTGAGAAC | TGGTGAAGACGCCAGTGGA | 138 |

**
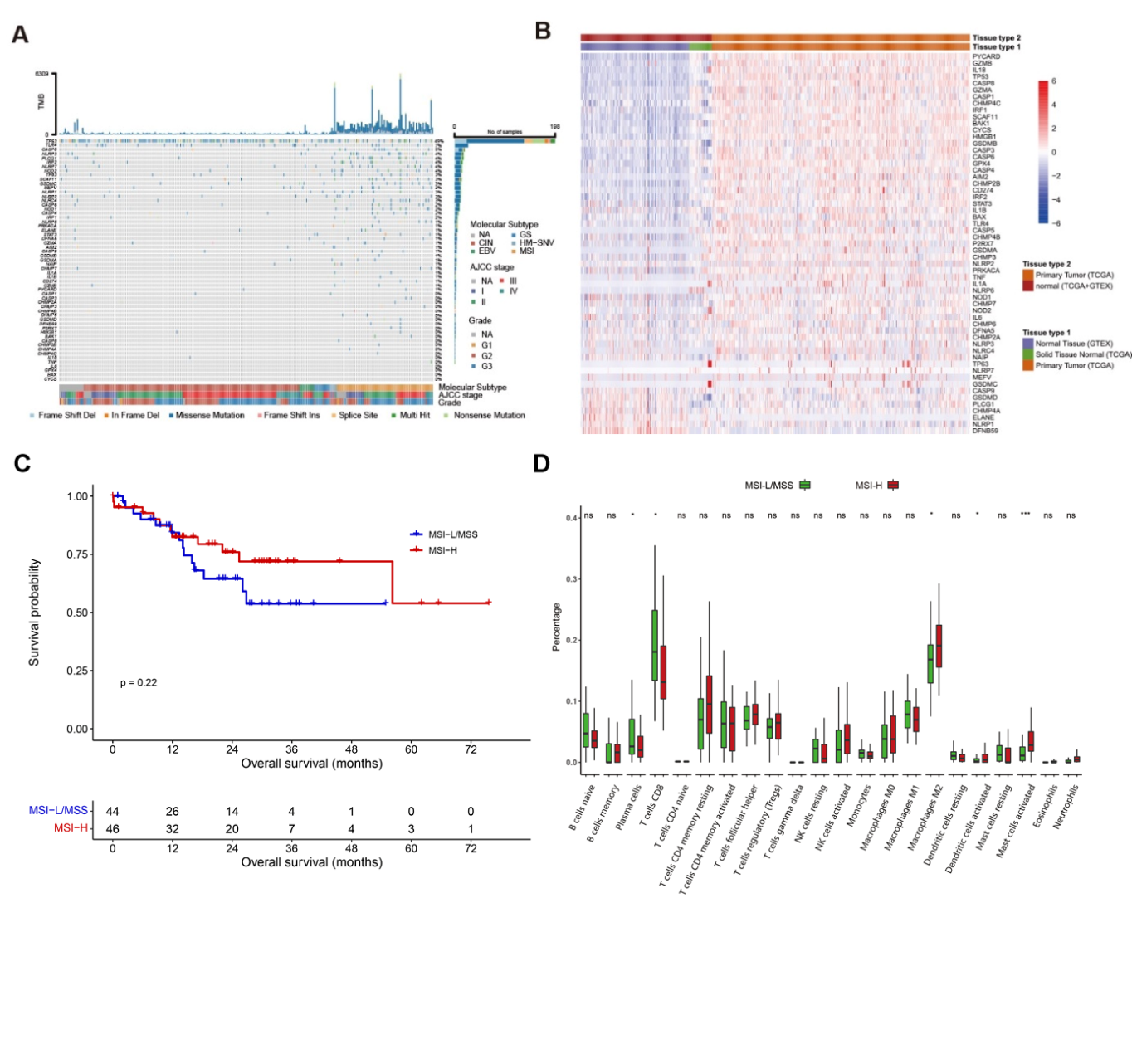
**

**Figure S1. Mutation and mRNA expression of 57 PRGs in gastric cancer.** (A) Mutation of 57 PRGs in the TCGA gastric cancer cohort. (B) mRNA expression of 57 PRGs between gastric cancer and normal gastric tissue in the TCGA and GTEX cohorts. (C) Kaplan-Meier curves of overall survival of Cluster 3 patients with MSI-H or MSI-L/MSS. (D) Proportion of the immune cell fractions in Cluster 3 with MSI-H or MSI-L/MSS. PRGs, pyroptosis-related genes. MSI-H, microsatellite instability-high. MSI-L/MSS, microsatellite instability-low/ microsatellite stable.

**
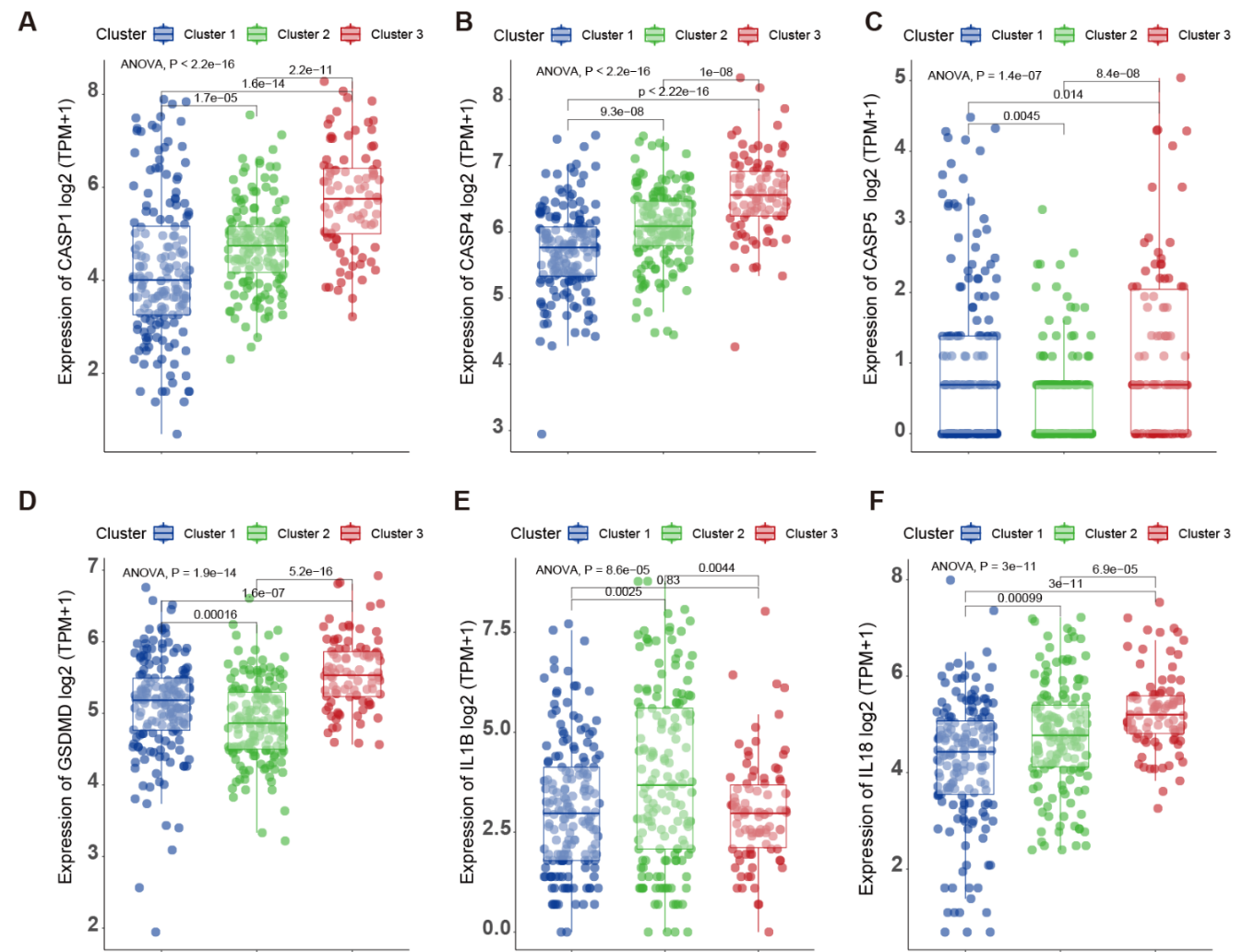
**

**Figure S2. Correlation between pyroptosis clusters and pyroptosis core genes.**

**
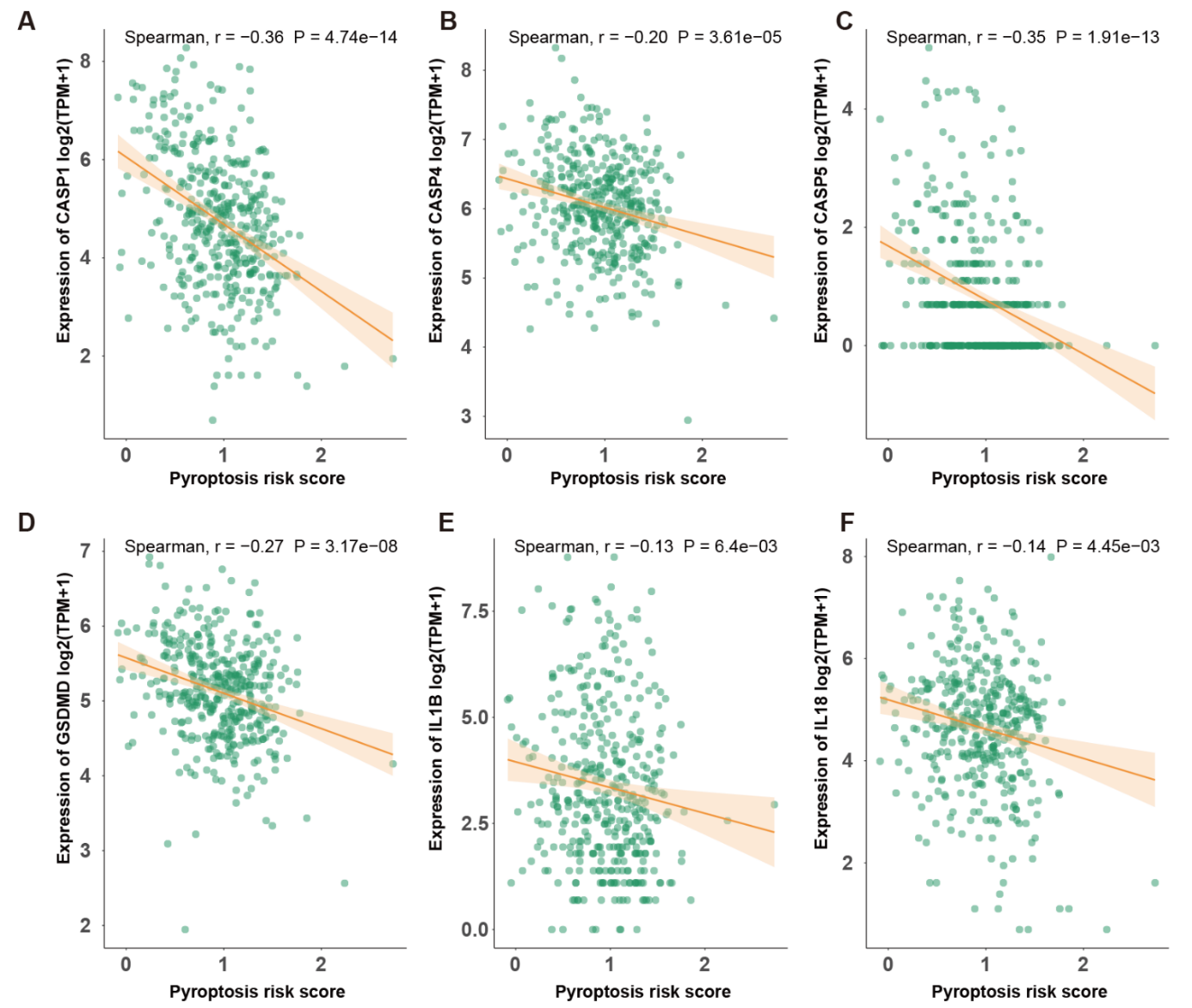
**

**Figure S3**. **Correlation between pyroptosis risk score and pyroptosis core genes.**


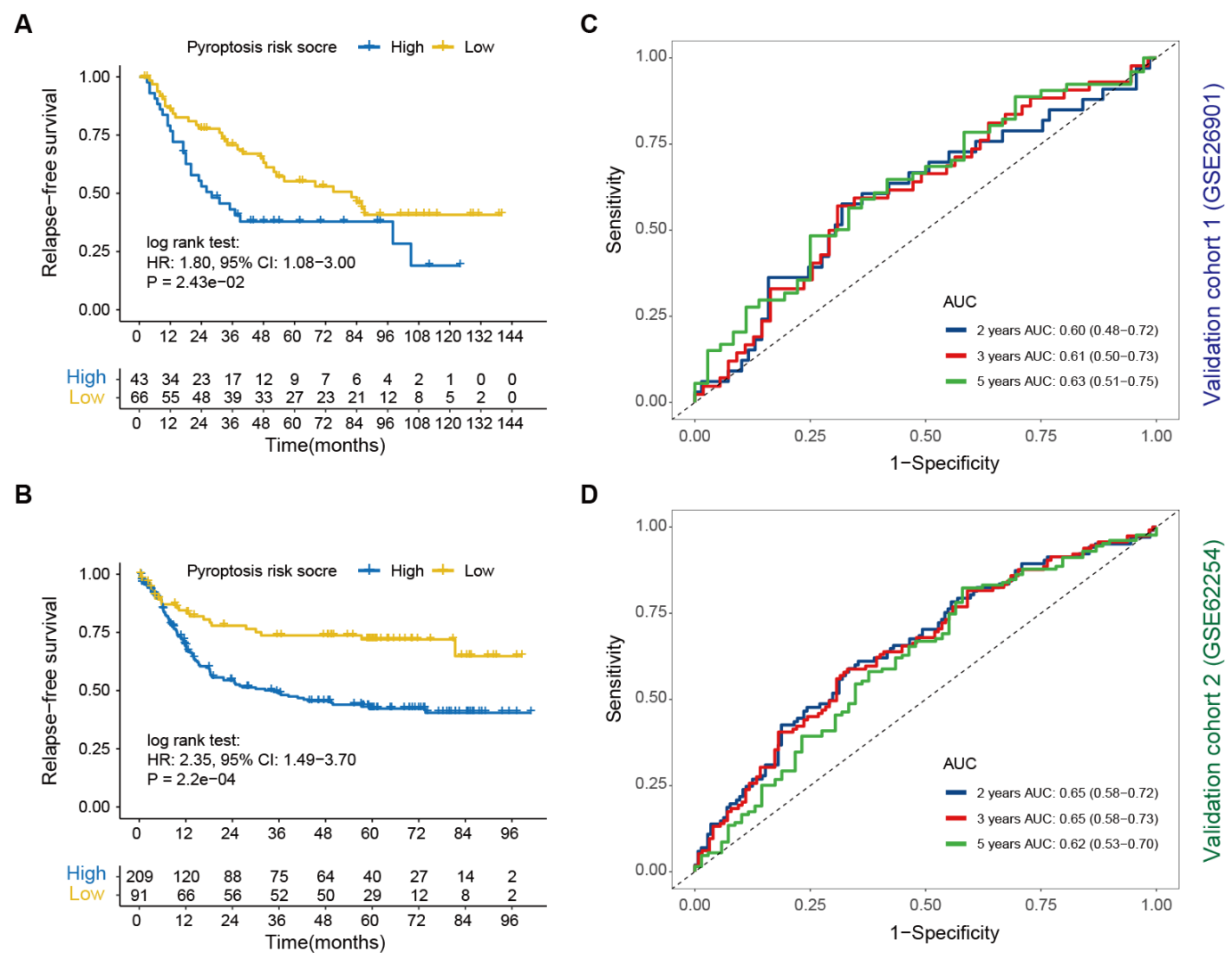


**Figure S4**. **Prognostic values of pyroptosis risk score for RFS in gastric cancer.** Kaplan-Meier curves of RFS by pyroptosis risk score in the validation cohort-1 (GSE26901; A) and validation cohort-2 (GSE62254; B). Time-dependent receiver ROC for 2, 3, 5 years RFS based on pyroptosis risk score in the validation cohort-1 (C) and validation cohort-2 (D). RFS, recurrence-free survival; ROC, receiver operating characteristics curves.

**
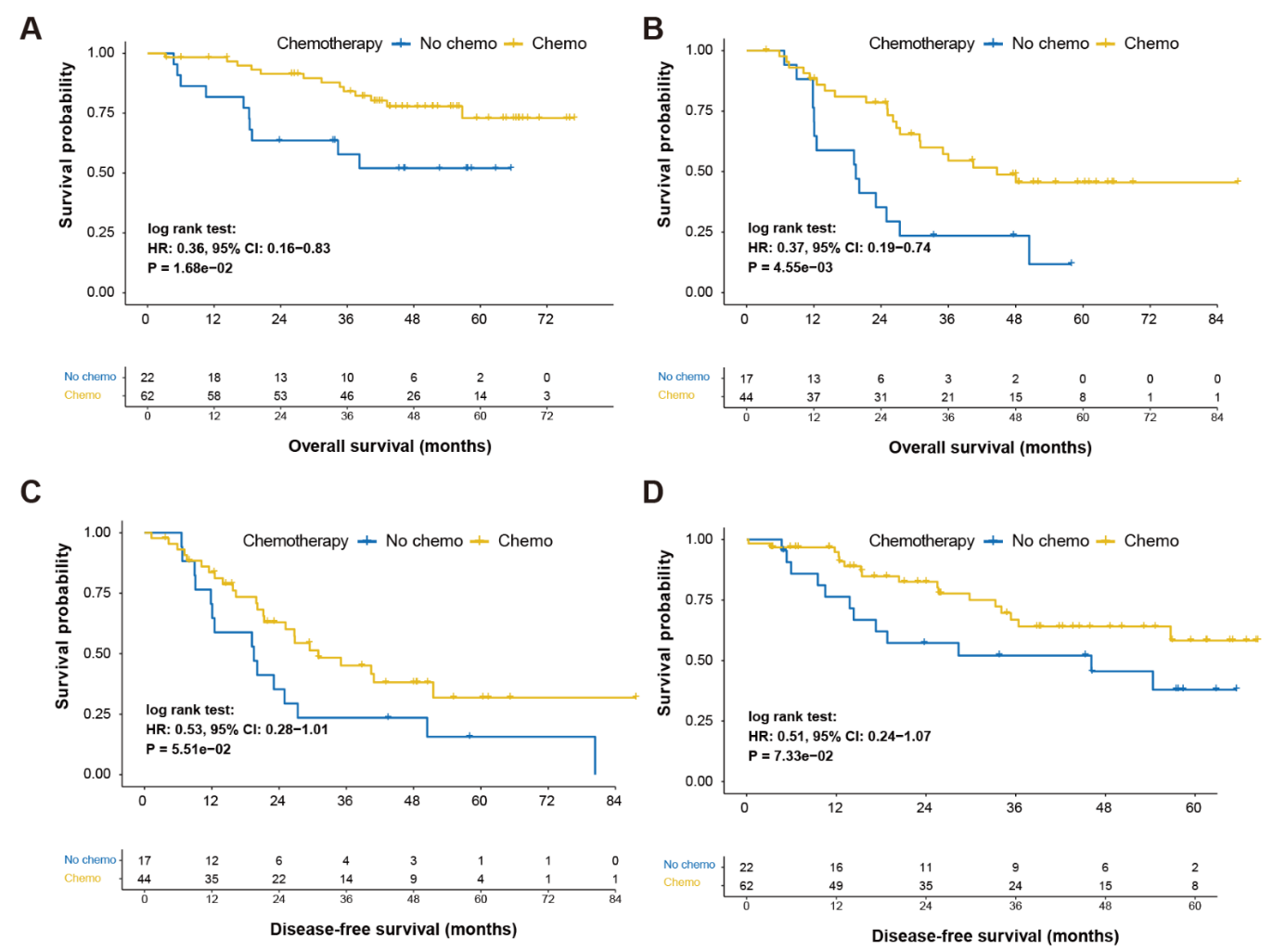
**

**Figure S5**. **Prognostic values of adjuvant chemotherapy stratified by pyroptosis risk score in the SYSUCC cohort.** Kaplan-Meier curves of overall survival by adjuvant chemotherapy in the high- (A) and low- (B) pyroptosis risk score of SYSUCC cohort. Kaplan-Meier curves of disease-free survival by adjuvant chemotherapy in the high- (C) and low- (D) pyroptosis risk score of SYSUCC cohort. SYSUCC, Sun Yat-Sen University Cancer center.
